# Advanced oral drug delivery systems for gastrointestinal targeted delivery: the design principles and foundations

**DOI:** 10.1186/s12951-025-03479-8

**Published:** 2025-05-30

**Authors:** Yafei Zhang, Yiran Wang, Yao Lu, Heng Quan, Yuqi Wang, Sijia Song, Huiyuan Guo

**Affiliations:** 1 College of Food Science and Nutritional Engineering, China Agricultural University, Beijing, 100083 China; 2 Key Laboratory of Precision Nutrition and Food Quality, Department of Nutrition and Health, China Agricultural University, Beijing, 100089 China; 3 Key Laboratory of Functional Dairy, College of Food Science and Nutritional Engineering, China Agricultural University, Beijing, 100083 China

**Keywords:** Biological barrier, Carrier, Targeting, Gastrointestinal tract, Inflammatory bowel disease

## Abstract

Oral administration has long been considered the most convenient method of drug delivery, requiring minimal expertise and invasiveness. Unlike injections, it avoids discomfort, wound infections, and complications, leading to higher patient compliance. However, the effectiveness of oral delivery is often hindered by the harsh biological barriers of the gastrointestinal tract, which limit the bioaccessibility and bioavailability of drugs. The development of oral drug delivery systems (ODDSs) represents a critical area for the advancement of pharmacotherapy. This review highlights the characteristics and precise targeting mechanisms of ODDSs. It first examines the unique properties of each gastrointestinal compartment, including the stomach, small intestine, intestinal mucus, intestinal epithelial barrier, and colon. Based on these features, it outlines the targeting strategies and design principles for ODDSs aimed at overcoming gastrointestinal barriers to enhance disease treatment. Lastly, the review discusses the challenges and potential future directions for ODDS development, emphasizing their importance for advancing drug delivery technologies and accelerating their future growth.

**Graphical Abstract:**

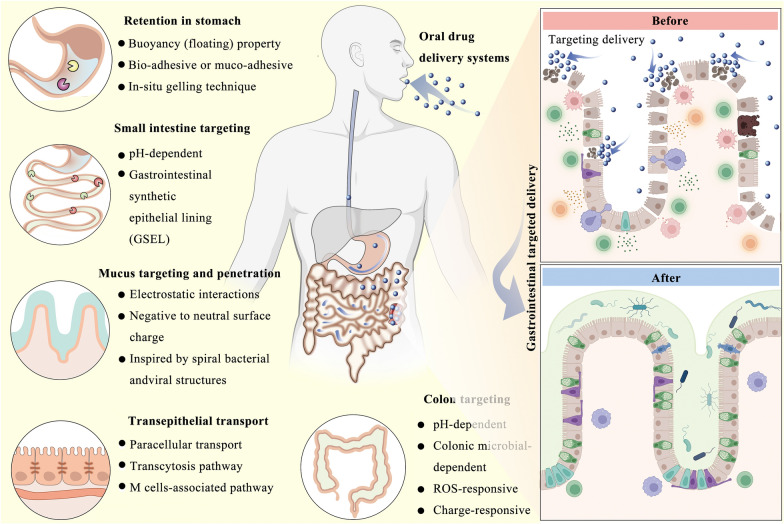

## Introduction

The development of a new drug involves substantial investment and extensive research before it can be approved for disease treatment. Drugs must undergo multiple stages of screening and validation, including discovery, lead optimization, Absorption, Distribution, Metabolism, Excretion, and Toxicity (ADMET) assessments, clinical trials, and approval via a New Drug Application (NDA) [1]. Only approximately 1 in 10,000 drug candidates are approved, while the majority are discarded. Consequently, the cost of bringing a drug molecule to clinical use can exceed $100 million [2]. Some promising candidates also face significant limitations, such as hydrophobicity, sensitivity to environmental factors like light, heat, and pH, and susceptibility to degradation [3]. Approximately 40–70% of drug candidates fail to reach sufficient therapeutic concentrations due to poor solubility and inadequate oral absorption. This issue is particularly pronounced for proteins and peptides, which, due to their large molecular size, struggle to penetrate biological barriers, resulting in very low oral bioavailability [4]. Based on solubility and membrane permeability, drugs are classified into four categories according to the Biopharmaceutical Classification System (BCS): Class I (high solubility, high permeability), Class II (low solubility, high permeability), Class III (high solubility, low permeability), and Class IV (low solubility, low permeability) [5]. The advancement of drug delivery systems has significantly improved the bioavailability of Class II, III, and IV drugs. These systems enhance drug stability in circulation, increase resistance to harsh environments, improve solubility, target specific organs more effectively, and minimize side effects on non-target organs, thereby optimizing pharmacodynamics and pharmacokinetics [3, 6]. Therefore, the drug delivery system is as crucial as the drug itself in ensuring therapeutic efficacy.

**Fig. 1:**
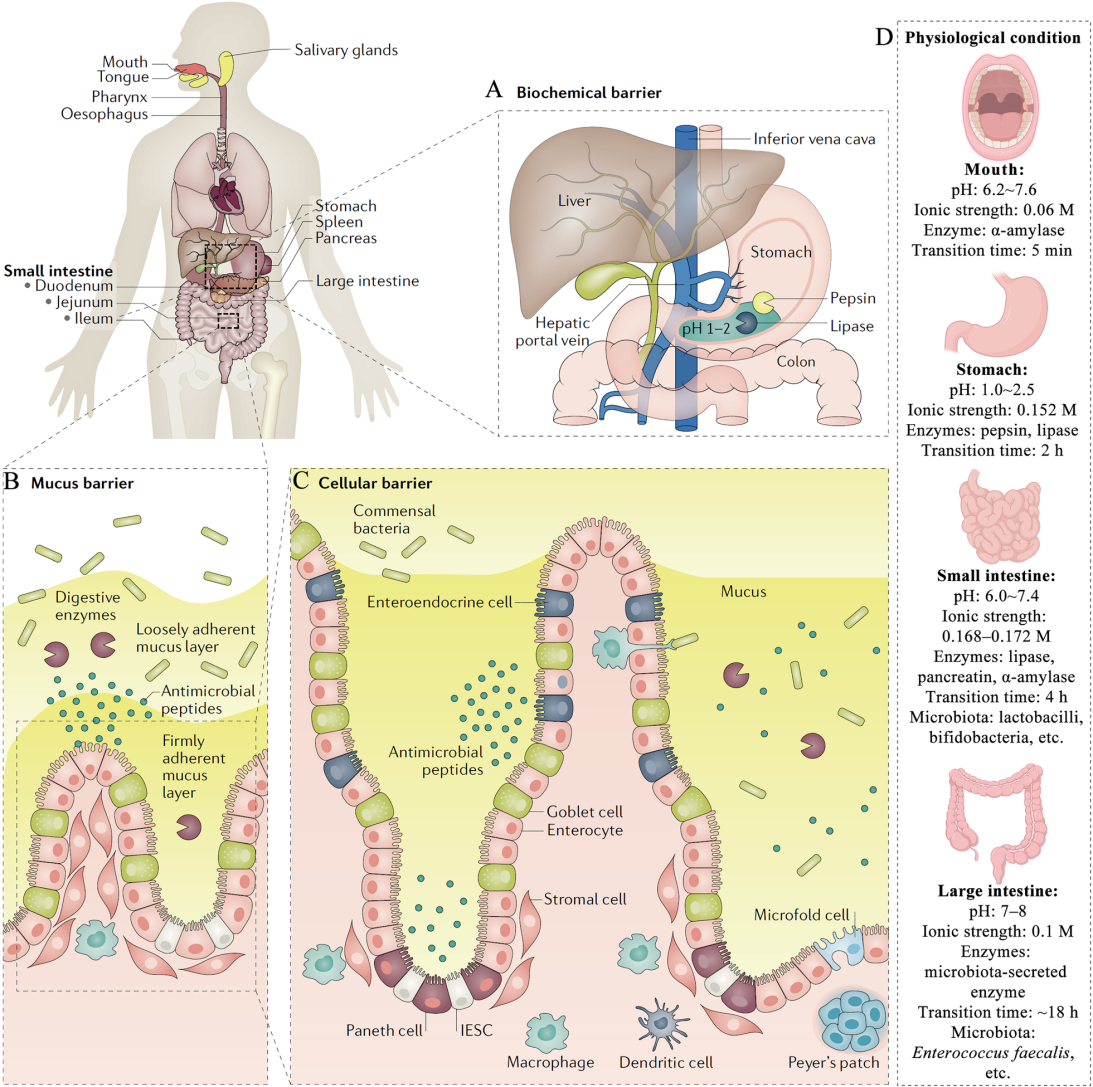
Physiological barriers to ODDSs [4]. Reproduced with permission [4]. Copyright 2020, Springer Nature. **A** Biochemical barrier: The combination of acidic/alkaline conditions and various enzymes in the GIT creates a complex environment that presents challenges for the stable utilization of ODDSs. **B** Mucus barrier: Mucus lines the entire GIT, acting as a physiological barrier between the epithelial cells and the lumen. **C** Epithelial barrier: This barrier controls the transport of nutrients between the intestinal lumen and the lymphatic system or bloodstream, but it also hinders the absorption of drug delivery systems. **D** Physiological conditions of the mouth, stomach, small intestine, and large intestine, including pH, ionic strength, enzymes, transition time, and microbiota [3]

Currently, injectable drugs are widely utilized for disease treatment. For instance, a majority of drugs approved by the U.S. Food and Drug Administration (FDA) are administered intravenously to alleviate patients' conditions [7]. While injections offer high bioavailability, with some achieving up to 80%, they are accompanied by numerous unavoidable side effects, including pain, skin infections, scarring, allergic reactions, and complications at the injection site [8, 9]. This is particularly problematic for patients with chronic diseases and cancers, such as diabetes, who require long-term treatment and monitoring. For these patients, the compliance and acceptability of injections are often poor. Additionally, intravenous injections require skilled healthcare professionals, and self-injections may lead to social stigma for patients [7]. In contrast, oral administration offers a non-invasive alternative, avoiding the discomfort associated with injections and significantly reducing both the economic burden and time required for treatment. However, oral administration faces significant challenges due to multiple biological barriers, such as biochemical, mucus, and cellular barriers, as illustrated in Fig. 1 [4]. Two primary biochemical barriers in the gastrointestinal tract (GIT) include pH and enzymes. The acidic stomach environment, the neutral to weakly alkaline conditions of the intestines, and enzymes (such as pepsin, lipase, and microbiota-secreted enzymes) can denature or degrade drugs [10]. The mucus barrier, a viscoelastic hydrogel-like substance secreted by goblet cells, functions as a physical obstacle to drug absorption. The cellular barrier regulates the transport of nutrients between the intestinal lumen and the lymphatic system or bloodstream [11]. Advanced oral drug delivery systems (ODDSs) aim to combine the advantages of both drug delivery technologies and oral administration, enhancing the ability of drugs to cross gastric acid and intestinal mucosal barriers, improving biosafety and targeting, preventing premature elimination, enabling controlled release, and prolonging their residence time at disease sites [7, 12]. Despite their potential, ODDSs face significant challenges due to the complexity of GIT biological barriers and the intricate interactions between drugs and these barriers. Nevertheless, ODDSs remain an exciting and challenging area of research. The first polymer micelle-based nanomedicine, Genexol-PM, was introduced in South Korea in 2007. Furthermore, BIND-014, the first actively targeted nanomedicine, entered clinical trials in 2011. While ODDSs are still in their infancy and primarily confined to laboratory research, they represent a promising direction for future medicine development.

**Fig. 2:**
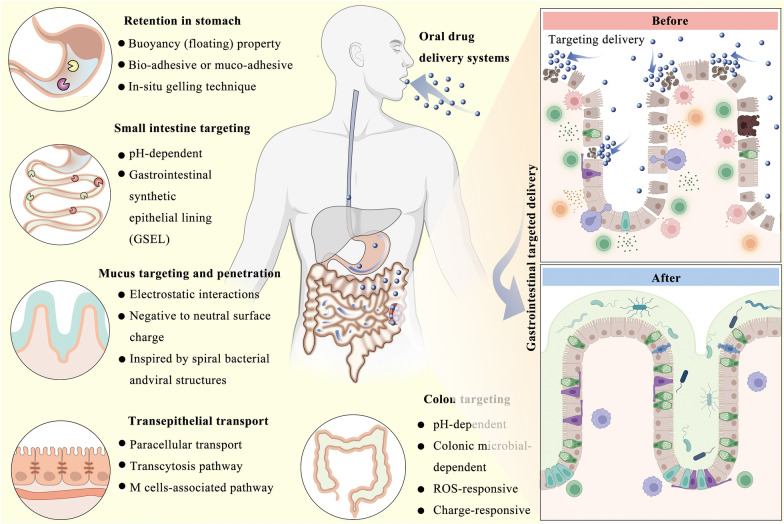
Advanced oral drug delivery systems for gastrointestinal targeted delivery: The design principles and foundations

This review outlines the physiological structure of the stomach, small intestine (including mucus and epithelial cell barriers), and large intestine (primarily the colon), along with the barriers that ODDSs encounter in these regions. Furthermore, the review systematically introduces the design principles and foundational concepts for ODDSs targeting specific gastrointestinal sites (Fig. 2). It also provides detailed examples of ODDSs developed for treating gastrointestinal-related diseases. Lastly, the challenges and potential future directions of ODDS development are discussed. The aim of this review is to inspire innovative designs and applications of ODDSs, ultimately benefiting patients.

## Stomach

### Physiological properties of the stomach

**Fig. 3:**
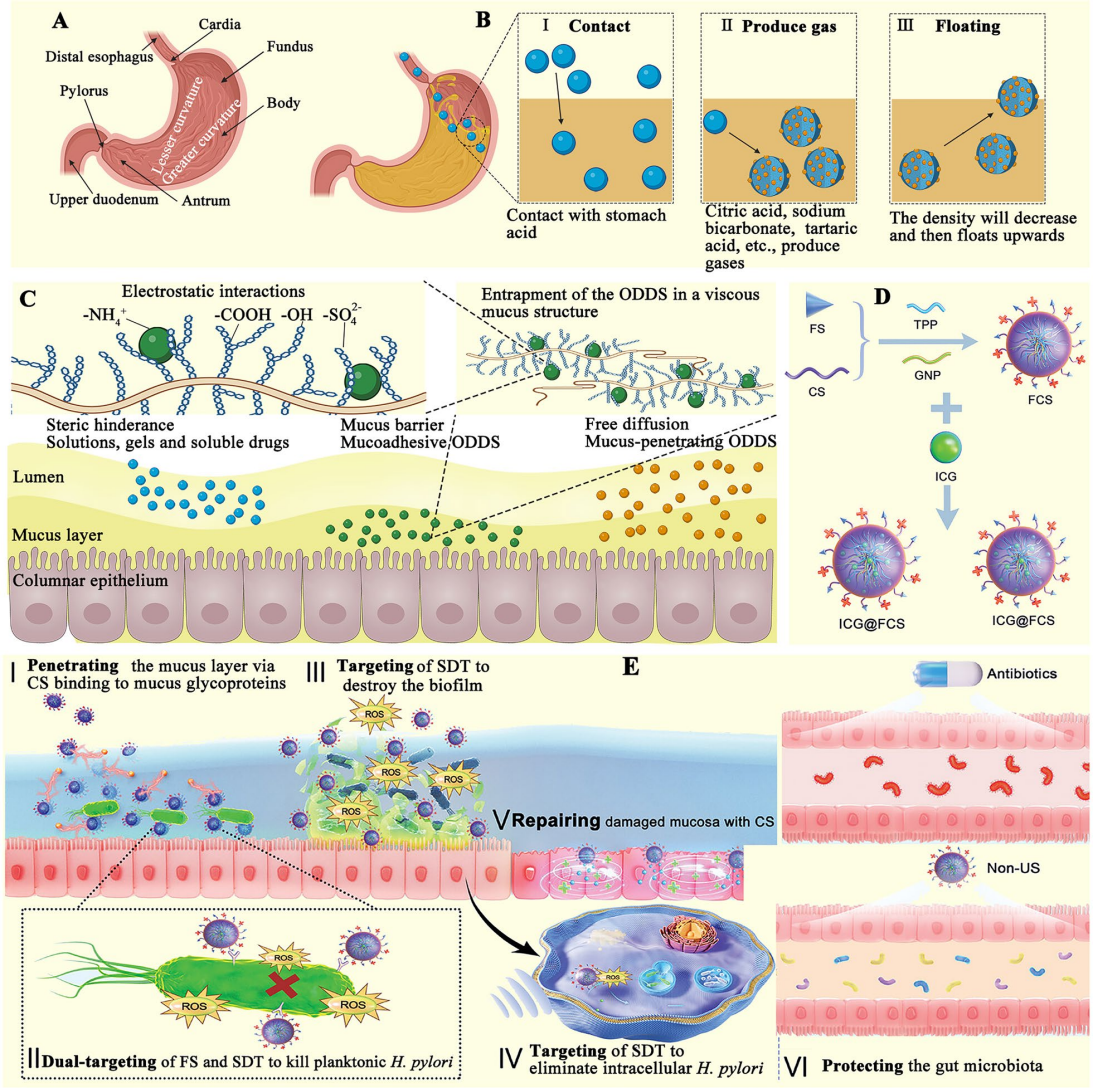
**A** Physiological structure of the stomach, including the distal esophagus, cardia, fundus, body, antrum, pylorus, upper duodenum, lesser curvature, and greater curvature [13]. **B** Effervescence mechanism: When various effervescent components (such as citric acid, sodium bicarbonate, and tartaric acid) come into contact with gastric acid, carbon dioxide is released through chemical reactions. This decreases the density of the ODDS, causing it to float upwards and maintain buoyancy for an extended period. **C** Mucoadhesion mechanism: The negatively charged sialic acid residues of mucin or negatively charged groups on cell surfaces (e.g., sulfate, carboxyl, and hydroxyl groups) can interact electrostatically with cationic groups, forming the primary adhesion mechanism. **D** Schematic of chitosan-conjugated fucose (FCS) and indocyanine green (ICG) @FCS preparation [22]. **E** Therapeutic activity of ICG@FCS: The ODDS (ICG@FCS) can penetrate the mucus layer, specifically recognizing and targeting *H. pylori*, generating reactive oxygen species under ultrasound stimulation. ICG@FCS exerts antimicrobial activity, eradicates biofilms, promotes autophagy to clear intracellular bacteria, and repairs gastric mucosa while protecting the intestinal flora [22]

The stomach is a highly deformable, crescent-shaped muscular pouch composed of several parts, including the antrum, cardia, body, fundus, pyloric sphincter, and pylorus, as depicted in Fig. 3A [13]. It plays a pivotal role in oral administration, with its primary function being digestion, which integrates mechanical forces, enzymatic degradation, and an acidic pH [14, 15]. After a meal, the stomach typically holds approximately 1.5 L of content, with fluctuations between 250 and 500 mL during digestion [16]. The release and absorption of drugs in the stomach are influenced by various factors. For instance, the stomach's highly acidic environment and the notably thick gastric mucus limit drug absorption. Additionally, the large volume of the stomach reduces the contact between the drug and gastric mucosa, resulting in a shorter retention time for the drug in the stomach [17]. Therefore, increasing the retention time of ODDSs in the stomach is a critical strategy for targeted release. The fundamental approach is to utilize the density and geometric properties of ODDSs to prevent gastric emptying and prolong their retention time in the stomach [14].

### Gastro-retentive ODDS

#### Buoyancy (floating) property of ODDS in gastric juice

The density of gastric juice ranges from 1.004 to 1.010 g/mL. If the density of an ODDS is lower than this, the system will float on the gastric juice, extending its retention time in the stomach [18]. To improve the floating behavior, effervescence is often incorporated into the system. Common effervescent components include citric acid, sodium bicarbonate, and tartaric acid [19, 20]. Upon contact with stomach contents, these effervescent agents react chemically to release carbon dioxide, which is trapped within the ODDS [18]. This bubbling and swelling mechanism reduces the density of the ODDS, causing it to float and maintain buoyancy for an extended period (Fig. 3B). For instance, in Yuan et al.'s study, sodium bicarbonate and hydroxypropyl methyl cellulose (HPMC) were used as the flotation agent and precipitant, respectively. This delivery system produced carbon dioxide to create a porous structure, reducing the true density of the system. They successfully controlled the floating time, floating lag time, and drug-release behavior using a continuous network structure matrix made from shellac (a green amphiphilic resin) [21]. The shellac-based system (containing 10 wt% sodium bicarbonate and 3 wt% HPMC) floated within 3 min and could remain buoyant for up to 16 h [21]. In addition to single-layer systems, double- and triple-layer ODDS designs are being explored to combine drugs with distinct release profiles. For example, one layer can be configured as a slow-release unit containing a gas-generating component, while the outer layer may contain another drug for rapid release [20].

#### Bio-adhesive or muco-adhesive

**Fig. 4:**
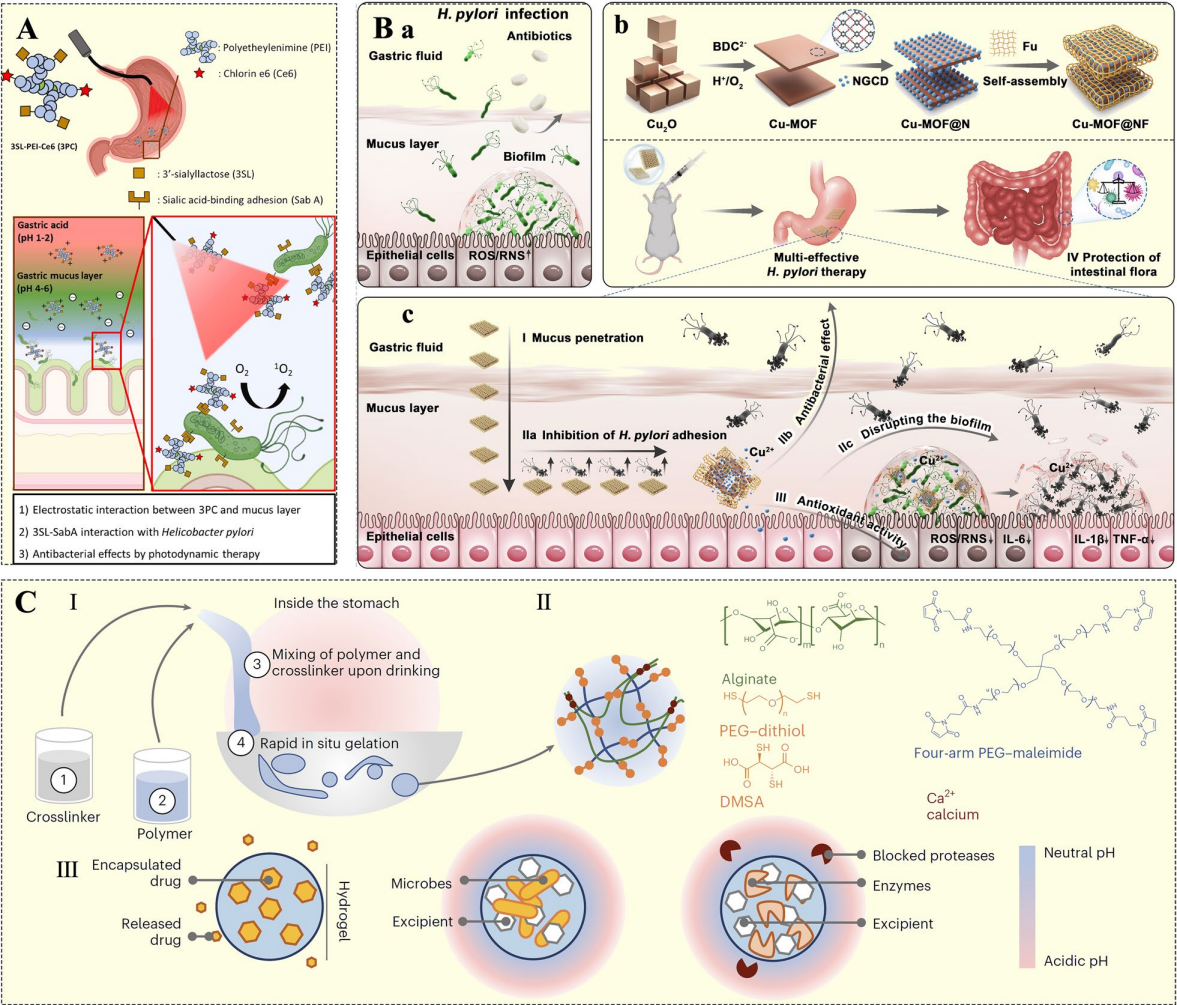
**A** Schematic illustration of the electrostatic interaction between the mucus layer and the 3′-sialyllactose-polyethyleneimine-chlorine e6 conjugate (3PC). 3′-sialyllactose exhibits specific binding affinity for *H. pylori* surface proteins, while chlorine e6, a photosensitizer, generates oxygen radicals in response to specific wavelengths [24]; Adapted with permission [24]. Copyright 2024, Elsevier. **B** a: The process of *H. pylori* infection of the gastric mucosa; b: The fabrication process of Cu-metal-organic framework (MOF) @NF (NF: nitrogen-doped carbon dots and naturally active polysaccharide fucoidan); c: Cu-MOF@NF can penetrate the gastric mucus layer, eliminate *H. pylori* planktonic bacteria and biofilms, regulate the inflammatory microenvironment, and maintain gut microbiota homeostasis [30]. **C** Through sequential ingestion of a crosslinker solution of dithiol crosslinkers and calcium, followed by a drug-containing polymer solution of four-arm poly(ethylene glycol)-maleimide and alginate, liquid in-situ-forming tough (LIFT) hydrogels form directly in the stomach [36]

The mucous layer in the stomach is a complex hydrogel primarily composed of water (95% w/w), mucin (less than 5% w/w), salts (1% w/w), cellular debris, carbohydrates, lipids, and antibodies [23, 24]. It serves as a selective barrier, effectively blocking gastric acid, preventing bacterial penetration, and preserving the integrity of the gastric epithelium [24]. Mucoadhesion involves multiple processes, primarily occurring in two stages: the contact stage and the consolidation stage. During the contact phase, the ODDS comes into close contact with the mucous layer, which leads to diffusion and subsequent swelling [25]. The peristaltic movements of the stomach can significantly impact mucoadhesion. In the contact stage, the delivery system encounters and overcomes various repulsive and attractive forces [26]. In the consolidation stage, moisture activates the mucoadhesive particles. Initially, moisture plasticizes the delivery system, allowing it to bind to the mucous membranes via weak bonds. To form a strong adhesive bond to the mucosal layer, the system must overcome the anti-adhesion properties of the mucosa [25]. This stage is explained by two main theories: diffusion theory and dehydration theory [27]. In diffusion theory, the mucosal adhesion delivery system and glycoproteins interpenetrate, forming non-covalent interactions through their respective chains. In dehydration theory, when materials come into contact with other gels in an aqueous environment, gelation occurs, and water is expelled until equilibrium is achieved [26]. This process is driven by osmotic pressure differences. Notably, the negatively charged sialic acid residues in mucins, or the negatively charged groups on cell surfaces (such as sulfate, carboxyl, and hydroxyl groups), can interact electrostatically with cationic groups, forming the primary adhesion mechanism (Fig. 3C) [28]. Thus, incorporating cationic substances into ODDSs can enhance their adhesive properties. Chitosan, in particular, is a prominent cationic substance [30, 31]. Yin et al. used chitosan and fucose, two non-toxic polysaccharides, to covalently bind indocyanine green (Fig. 3D) [22]. Due to the cationic nature of chitosan, this delivery system effectively binds to the anionic gastric mucus, improving adhesion and penetration to the gastric mucosa. Moreover, fucose specifically targets the lectin receptor on *Helicobacter pylori* (*H. pylori*), providing a molecular targeting mechanism. Upon ultrasound activation, indocyanine green generates singlet oxygen (^1^O_2_), which aids in eliminating biofilms of floating bacteria while inducing autophagy to remove intracellular pathogens. These targeting mechanisms ensure precise delivery to *H. pylori*, contributing to gastric mucosal repair (Fig. 3E) [22]. Additionally, polylysine and polyethylenimine (PEI), both containing abundant -NH_2_ groups, are effective adhesion enhancers. For example, a 3'-sialyllactose-PEI-chlorin e6 conjugate (3PC) was synthesized [24]. The cationic polymer PEI extends the retention time of ODDSs in the stomach, increasing the likelihood of encountering *H. pylori* and thereby enhancing the antibacterial efficacy of the system (Fig. 4A) [24]. *H. pylori* possesses sialic acid-binding adhesin in its outer membrane proteins, enabling it to specifically bind to ɑ-2,3-linked terminal sialic acid (e.g., 3'-sialyllactose) on the sialyl-dimeric-Lewis structure. This interaction facilitates the precise targeting of *H. pylori* through ɑ-2,3-linked terminal sialic acid 3'-sialyllactose [24].

In addition to electrostatic interaction (EI), hydrogen bonding (H-bond) and hydrophobic interactions significantly contribute to the adhesion process. Hydrogen bonds are permanent dipole forces that typically occur between a covalently bound hydrogen atom and a more electronegative atom (such as O, N, or C), interacting with another electronegative atom [3]. Mucin contains both hydrogen donors and acceptors, facilitating H-bond formation. Certain regions of stomach mucin glycoproteins exhibit hydrophobic characteristics, leading to hydrophobic interactions when they approach hydrophobic fragments in the delivery system. The hydrophilicity of the system’s components aids the ODDS in penetrating the gastric mucus layer. Fucoidan, a naturally occurring polysaccharide, demonstrates excellent biocompatibility, stability, and hydrophilicity, enabling the delivery system to adhere to and penetrate the gastric mucous layer effectively [29]. Shu et al. encapsulated nitrogen-doped carbon dots with copper-containing metal–organic frameworks (Cu-MOFs), which were then coated with fucoidan (Fig. 4B) [30]. The results indicated that this system successfully penetrated the mucous layer, inhibiting *H. pylori* adhesion to gastric epithelial cells. The released Cu^2+^ ions degraded biofilm polysaccharides and disrupted the cyclic growth pattern of bacterioplankton ↔ biofilm, thereby preventing recurrence and persistent infection [30].

#### In-situ gelling technique

The in-situ gelling technique, also known as the raft-forming system, represents an intermediate state between liquid and solid components [31]. Hydrogels, which are three-dimensional structures, possess the ability to swell and retain significant amounts of water and biological fluids. In-situ gels are hydrogels in solution form that undergo gelation upon contact with gastric acid [32]. Formulations typically include gellan gum or sodium alginate solutions containing calcium chloride and sodium citrate. When exposed to gastric acid, calcium ions are released, and gellan gum or sodium alginate acts as a gelling agent. The calcium ions become trapped within the polymer chains, facilitating cross-linking and forming a matrix structure [33]. This gelation process involves the formation of double helix connections, which are further reassembled into a three-dimensional network structure through cationic complexation and hydrogen bonding with water [34]. Additional polymers that can form in-situ gels include xyloglucan, chitosan, and polycarolactone [33]. In Fan et al.'s study, deacetylated gellan gum, sodium alginate, calcium citrate, and Bletilla striata polysaccharide (BSP) were used to achieve sustained release of BSP. The in-situ gel exhibited excellent fluidity in vitro and self-assembled in the acidic environment of pH 1.5. The extrusion, deformation, and shear properties increased by 103.7, 46.3, and 26.4%, respectively. Furthermore, the retention time in the stomach was extended up to 4 h [35]. A recent study demonstrated that liquid in-situ-forming tough (LIFT) hydrogels could form directly in the stomach by continuously ingesting a crosslinker solution of dithiol crosslinkers and calcium, followed by ingestion of a polymer solution containing four-arm poly(ethylene glycol)-maleimide and alginate (Fig. 4C) [36]. Additionally, some pH-sensitive polymerizations can interact with each other to form in-situ gels in acidic gastric environments. These polymers contain acidic or alkaline groups that accept or release protons depending on the pH of the environment. In alkaline environments (above the pKa), polymers with weakly basic groups, such as poly(methacrylic acid) containing carboxylic acid, become deprotonated and acquire a negative charge [32]. This results in increased electrostatic repulsion between polymer molecules, leading to changes in physical morphology. Therefore, the formation of in-situ gels is influenced by polymer-to-polymer interactions. For example, hydrogen bonding between carboxyl pairs can promote gel formation at pH values below the pKa. When the pH exceeds the pKa, repulsion between the polymers occurs, resulting in a more fluid solution [37]. Notably, gastric-targeted ODDSs often combine multiple retention strategies to enhance targeting efficacy. For example, bicarbonate may be added to the system to increase buoyancy by releasing carbon dioxide, which produces effervescence and reduces the gel's density [38]. Other representative experiments for gastric targeting are summarized in Table 1.

**Table 1:** Representative experiment of oral drug delivery systems for gastrointestinal targeted delivery

Target site	Disease type	Building blocks	Cargoes	Targeting mechanism	Therapeutic effect	References
Stomach	Gastritis	Polyvinylpyrrolidone, Fe^3+^	Edible natural active polyphenols (curcumin, quercetin, and fisetin)	pH-responsive	Nanozymes reduce intracellular oxidative stress by efficiently scavenging ROS, thereby minimizing oxidative damage. Furthermore, in inflammatory environments, nanozymes can also lower the expression of pro-inflammatory factors, effectively modulating immune cell function and achieving precise regulation of inflammatory responses	[203]
Stomach	*H. pylori* infection	Mesoporous organosilica nanoparticle (MON), hyaluronic acid (HA), bacterial outer membrane vesicle (OMV)	Clarithromycin (CLR), Ag^+^	pH-responsive	OMVs encapsulated in the outer layer of the ODDS offer cytoprotective and anti-inflammatory effects at the site of inflammation, further improving biosafety and therapeutic efficacy. In cases of *H. pylori* infection in the stomach, the delivery system harnesses the synergistic effects of Ag^+^ and CLR to effectively eradicate drug-resistant *H. pylori*, surpassing the antimicrobial efficacy of clinically used triple therapy	[204]
Stomach	*H. pylori* infection	Poly(lactic-co-glycolic acid) (PLGA), Chitosan	Clarithromycin (CLR)	Electrostatic interactions	The ODDS can efficiently load clinical doses of the drug, remain in the mouse stomach wall, and exhibit significant bactericidal activity in vivo. It has been demonstrated that this ODDS can effectively treat *H. pylori* infection without the need for commonly used proton pump inhibitors (PPIs), thereby avoiding side effects such as headache and diarrhea typically associated with PPI use during treatment	[205]
Stomach	*H. pylori* infection	Chitosan	Polydopamine, zinc oxide, silver nitrate	Electrostatic interactions	In a mouse model of *H. pylori* infection, the ODDS actively targeted submucosa-colonized *H. pylori* by exploiting gastric mucus permeability, effectively eradicating both the bacteria and its biofilms through ROS generation. Transcriptomic analysis revealed no significant side effects on the gut microbiota during treatment	[206]
Stomach	*H. pylori* infection	Rhamnolipids (RHL)	Berberine derivatives (BD)	RHL shielded the positive charge of BD and increased the hydrophilicity	The ODDS not only demonstrated a strong ability to eliminate *H. pylori* biofilms but also inhibited the adhesion of *H. pylori* to biological surfaces, effectively disrupting the critical steps involved in infection recurrence	[207]
Small intestine	Infection of conditioned pathogens	Glucose, polylactic acid-glycolic acid polymer	Antibiotics (ampicillin, chloramphenicol)	Ligand-receptor specific binding	The ODDSs significantly enhance the delivery efficiency of antibiotics, facilitating their entry into the bloodstream and increasing bioavailability. This, in turn, enables effective treatment of infections caused by intestinal-conditioned pathogens	[208]
Small intestine	Gout	Alginate	Uricase, dopamine	pH-responsive	Gut-targeted hydrogel microspheres immobilized uricase in the intestinal mucosa, effectively inhibiting gout by regulating uric acid excretion via intestinal transporters	[209]
Small intestine	Diabetes mellitus	*C. vulgaris* (CV), alginate (ALG), calcium chloride	Insulin	pH-responsive	Oral insulin-loaded carriers demonstrated more effective and sustained glucose-lowering effects compared to direct insulin injections, without causing any damage to the intestines	[52]
Small intestine	Deliver nutrients	Alginate, dopamine	Amoxicillin, levodopa	Covalent interactions	The development of mucoadhesive ODDSs can protect both hydrophilic and hydrophobic drugs during continuous oral administration. With careful optimization, these systems can load a range of drugs at high doses and prolong the duration of drug retention in the body, thereby reducing the frequency of medication intake	[61]
Small intestine	Deliver nutrients	Alginate	Six essential mineral elements	pH-responsive	Microcapsules are designed to effectively protect encapsulated nutrients, such as essential mineral elements, from the acidic environment of the stomach and deliver them precisely to the small intestine	[210]
Mucus	Colorectal cancer	Chitosan	Indocyanine green derivative	Ligand-receptor specific binding	Oral hydrogel-based combination therapy with immune checkpoint inhibitors can inhibit the growth of primary and distal tumors by directly killing tumor cells, reversing the immunosuppressive tumor microenvironment, and enhancing systemic anti-tumor immunity	[211]
Mucus	Deliver nutrients	Pea albumin	Capsaicin (CAP)	pH-responsive	Pea albumin nanomicelles represent a promising oral drug delivery system capable of effectively overcoming the harsh gastrointestinal environment and mucus barrier, thereby enhancing intestinal absorption and bioavailability of hydrophobic bioactives	[212]
Mucus	Ulcerative colitis (UC)	Polyethylene glycol (PEG)	Probiotics	Electrostatic interactions	Strengthening the intestinal mucosal barrier through oral administration of commensal bacteria coated with PEG promotes their penetration into the mucus. This approach not only inhibits the invasion of pathogenic bacteria and maintains intestinal flora homeostasis but also stimulates mucus secretion and the expression of tight junctions (TJs), preventing the development of colitis and diabetes in mice	[70]
Mucus	IBD	Monomer diiodobutadiyne (IDA)	Iodine	Adhere to the mucus layer (similar to bacterial flagella)	PIDA nanofibers are highly effective in alleviating inflammation while maintaining intestinal microbiota homeostasis, thereby promoting the rebalancing of the gastrointestinal microenvironment and facilitating mucosal healing	[213]
Mucus	IBD	Polyacrylic (PAA)	Ceria nanoparticles (CeNPs)	Electrostatic interactions	Antioxidant nanotherapeutics exhibit preferential adhesion to the inflamed epithelium and are distributed throughout the inflamed colon tissue, effectively regulating oxidative stress levels at the lesion sites. These properties make them promising candidates for the treatment of inflammatory bowel diseases	[214]
Colon	UC	β-cyclodextrin (CD), 4-(hydroxymethyl)phenylboronic acid (PAPE)	Celastrol (Cel)	ROS sensitivity Electrostatic interactions	Nanomedicine can effectively target the colon and release triptolide, alleviating ulcerative colitis (UC) by reducing inflammatory cytokines, scavenging ROS, and inhibiting colon cell apoptosis	[215]
Colon	UC	Ammonium chitosan (HTCC), succinic acid-modified γ-cyclodextrin (SACD)	Curcumin (Cur)	pH-responsive electrostatic interactions	Nanoparticles enhanced the oral bioavailability of curcumin (Cur) and provided more effective relief of colitis by restoring the intestinal barrier, modulating the inflammatory response, and maintaining colonic microbiota homeostasis	[216]
Colon	UC	Medium chain triglyceride, beeswax, chitosan, octenyl succinic-resistant starch	Cur	Enzyme responsiveness	The nanostructured carrier ensured the prolonged residence time of curcumin (Cur) in the colonic mucus and specifically targeted microbiota enzymes for degradation, thereby significantly improving the condition of colonic mucosal inflammation	[67]
Colon	UC	Hyaluronic acid (HA)	*Clostridium butyricum* (*C. butyricum*)	H-bond interactions	The nano-coating not only reduced microbial translocation but also protected *Clostridium butyricum* from gastrointestinal damage, thereby more effectively alleviating intestinal inflammation. In animal models of chronic kidney disease and acute kidney injury, the oral delivery of nanocoated bacteria demonstrated the ability to eliminate renal fibrosis and restore renal function	[217]
Colon	UC	Chitosan, Low–molecular weight heparin (LMWH)	ε-polylysine (EPL)	LMWH acts as an adhesion ligand for integrins α4 and αM	Oral nanotherapeutic agents, such as oxidation-sensitive EPL nanoparticles (OPNs)@LMWH, effectively treated mouse models of colitis by improving oxidative stress, inhibiting the activation of inflammation-related signaling pathways, and enhancing the protective mechanisms of the colon epithelium	[218]

#### Combination systems

Although gastro-retentive ODDSs can improve patient compliance and enhance drug bioavailability, each individual retention method has inherent limitations. For example, the floating system is highly dependent on the patient’s feeding state, the bio-adhesive system may be influenced by the continuous turnover of mucus, and the in-situ gelling technique may suffer from compromised integrity due to mechanical pressure [39]. To overcome these challenges, combining various individual systems can mitigate the limitations of a single method, enabling the delivery of drugs with narrow absorption windows. This approach not only enhances drug bioavailability but also reduces the frequency of administration [40]. This has become a prominent research focus in the development of new gastric retention technologies [40, 41]. For instance, Meng et al. developed a synergistic gastro-retentive drug delivery system combining floating and bio-adhesive properties [42]. The delivery efficiency of furosemide hollow-bio-adhesive microspheres was optimized using the Box-Behnken design. The in vitro flotation rate and retention rate within 8 h were 93 and 91%, respectively, while the drug’s sustained-release efficiency over 12 h reached 94%. Compared to hollow microspheres with only floating characteristics, the hollow-bio-adhesive microspheres significantly increased their retention time in the stomach. Moreover, when compared to commercially available tablets, these microspheres improved the relative bioavailability of furosemide by 164% [42]. In the experiment conducted by Nigusse et al., a gastric retention system featuring three key characteristics—expandability, floating, and bio-adhesion—was developed. The delivery system incorporated sodium carboxymethyl cellulose and hydroxypropyl methylcellulose (HPMC) as release-retarding polymers, microcrystalline cellulose as a direct compression diluent, and sodium bicarbonate as a gas-generating agent. The results revealed a floating lag time of 5.09 s, a biological viscosity strength of 29 g, and a 12 h swelling index of 315%. This gastro-retentive ODDS, combining expandability, floating, and bio-adhesive properties, effectively addressed the issues of low bioavailability, short biological half-life, and challenges with gastric targeting of Ranitidine HCl [43]. Furthermore, the in-situ gelling technique can be integrated with other gastro-retentive strategies. For instance, in Siripruekpong et al.'s study, in-situ gelling technology was combined with a floating system to create a gastro-retentive and raft-forming delivery vehicle [31]. The dispersion of resveratrol and curcumin (bioactive substances with poor water solubility and rapid metabolism) was first prepared by solvent evaporation technology with Eudragit^®^EPO, then mixed into an in-situ gelling liquid formulation containing sodium alginate, pectin, and carrageenan. The addition of sodium bicarbonate and calcium carbonate generated carbon dioxide bubbles in the gel matrix upon contact with gastric juice, enabling the system to float. Calcium ions also acted as crosslinking agents for the hydrogel, facilitating in-situ gel formation. In simulated gastric juice, this multi-component system achieved rapid floating and maintained buoyancy within one minute, gradually releasing over 80% of resveratrol and curcumin within 8 h [31]. These studies demonstrate that combining various gastric retention methods can result in more predictable and enhanced drug release profiles. This is due to the combined effects of gastric emptying processes and lag times, as a single system may leave the stomach before it can exert its effects [23]. However, another significant challenge in formulation design is maintaining simplicity. An overly complex design may introduce variability and limit the system's effectiveness in clinical application.

### Physiological properties of the small intestine

The small intestine, the longest and most complex segment of the GIT (approximately 6 m in length), is where digestion primarily occurs through enzymes secreted by the liver and pancreas. The presence of microvilli and villi significantly increases the surface area of the small intestine, expanding it to approximately 200 m^2^ in adults [44]. Villi, finger-like projections extending into the intestinal lumen, are covered by epithelial cells. Due to its extensive surface area and prolonged transit time, the small intestine serves as the primary site for drug absorption within the GIT. Additionally, the motility of the small intestine is more consistent than other parts of the GIT, making the performance of ODDSs more predictable and uniform. The primary aim of targeting the small intestine is to enhance ODDS absorption, achieve localized drug release to improve bioavailability, or optimize systemic drug delivery [45]. Unlike the stomach, the small intestine does not maintain a strongly acidic environment, especially in the distal regions, and some ODDSs leverage this elevated pH as a trigger for drug release. The small intestine also features more discontinuous and thinner mucus, enabling it to absorb a broader range of molecules. However, enzymatic degradation remains a concern for drug absorption, as it predominantly occurs in the duodenum, where enzymes facilitate the breakdown and absorption of lipids, proteins, and carbohydrates [46]. Consequently, the primary objective of ODDS targeting the small intestine is to safely transport the drug from the acidic and enzymatically active stomach environment to the small intestine, preserving the drug’s activity [47]. The main strategies for targeting the small intestine with ODDSs include pH-dependent enteric polymers and gastrointestinal synthetic epithelial linings (GSEL).

### Small intestinal-targeted ODDSs

#### pH-dependent

**Fig. 5:**
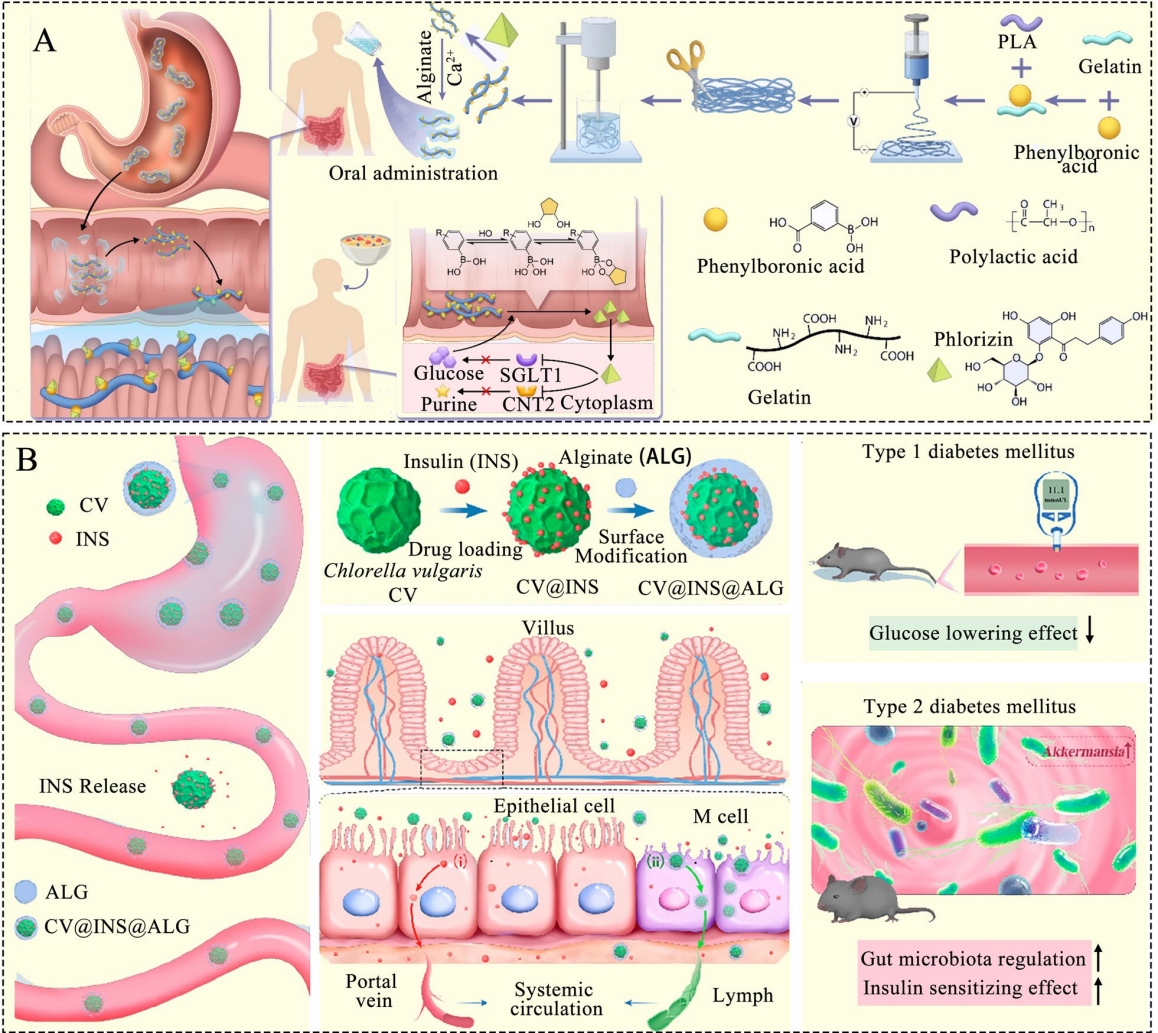
**A** Schematic illustration of the preparation procedure for intestine-settled electrospun short fibers, which reduce the absorption of glucose and purines. The electrospun short-fiber system was constructed using ion crosslinking and electrospinning technology. The oral short fibers pass through the stomach, where the calcium alginate shell breaks down, allowing the fibers to settle in the intestinal villi [49]; Adapted with permission [49]. Copyright 2025, Springer Nature. **B** A microalgae-based oral insulin (INS) delivery strategy developed using *Chlorella vulgaris* (CV)-based INS delivery system coated by sodium alginate (ALG). CV@INS@ALG protects INS from harsh gastric conditions (gastric acid and enzymes), overcomes the gastrointestinal barrier, and achieves pH-responsive drug release in the intestine [52]; Adapted with permission [52]. Copyright 2023, American Chemical Society

When ODDSs target the small intestine, the first biological barrier they encounter is the harsh gastric environment. The acidic conditions in the stomach (pH 1.0–2.5) can denature acid-sensitive compounds, while the abundant digestive enzymes rapidly degrade the cargo. Thus, protecting drug stability across the gastric barrier becomes a crucial consideration when targeting the small intestine [48]. Given the significant pH changes between the highly acidic stomach and the weakly alkaline intestinal tract, pH dependency serves as a major mechanism for intestinal targeting. ODDSs that respond to pH changes, altering their interaction with the surrounding environment, are termed pH-responsive [47]. These pH-responsive polymer systems are typically formulated with polycations, polyanions, or combinations and crosslinks of both. The release mechanism involves dissolution and expansion at a specific pH [47]. Calcium alginate is commonly used as a building block for releasing intestinal-targeted drugs. It remains stable in the gastric acidic environment but disintegrates in the neutral environment of the intestine [46]. To treat hyperuricemia and hyperglycemia, Tang et al. used gelatin and polylactic acid (PLA) to fabricate fibers through a refined electrospinning-homogenizing process (Fig. 5A) [49]. The outer layer of the delivery system was coated with calcium alginate, ensuring that pH changes in the intestine triggered the collapse of the system. In both diabetic and hyperuricemia mouse models, treatment with staple fibers reduced glucose levels by 17.8% and blood uric acid by 49.6% [49]. Proteins and peptides, such as insulin, are prone to degradation in the stomach. However, oral insulin administration is more convenient than subcutaneous injection, offering better patient compliance [50, 51]. To overcome the gastrointestinal barrier and target the small intestine for enhanced insulin absorption, Ren et al. developed pH-responsive microalgal hydrogels for oral insulin delivery [52]. The insulin-loaded system was coated with sodium alginate and gelled with calcium chloride. This system effectively protected insulin from degradation in the acidic gastric environment and released it in the intestinal pH [52].

Additionally, in the experiment conducted by Leong et al., carboxymethylation of kappa-carrageenan (a natural linear polysaccharide) was used to impart pH-dependent swelling properties, enabling the intestinal targeted delivery of bioactive macromolecules [53]. In simulated gastric juice, the release of the model drug was low (23%), while in simulated intestinal fluid, it was significantly higher (90%). Overall, the application of pH-dependent properties in small intestinal targeted delivery is quite widespread [51, 54].

#### Gastrointestinal synthetic epithelial lining

**Fig. 6:**
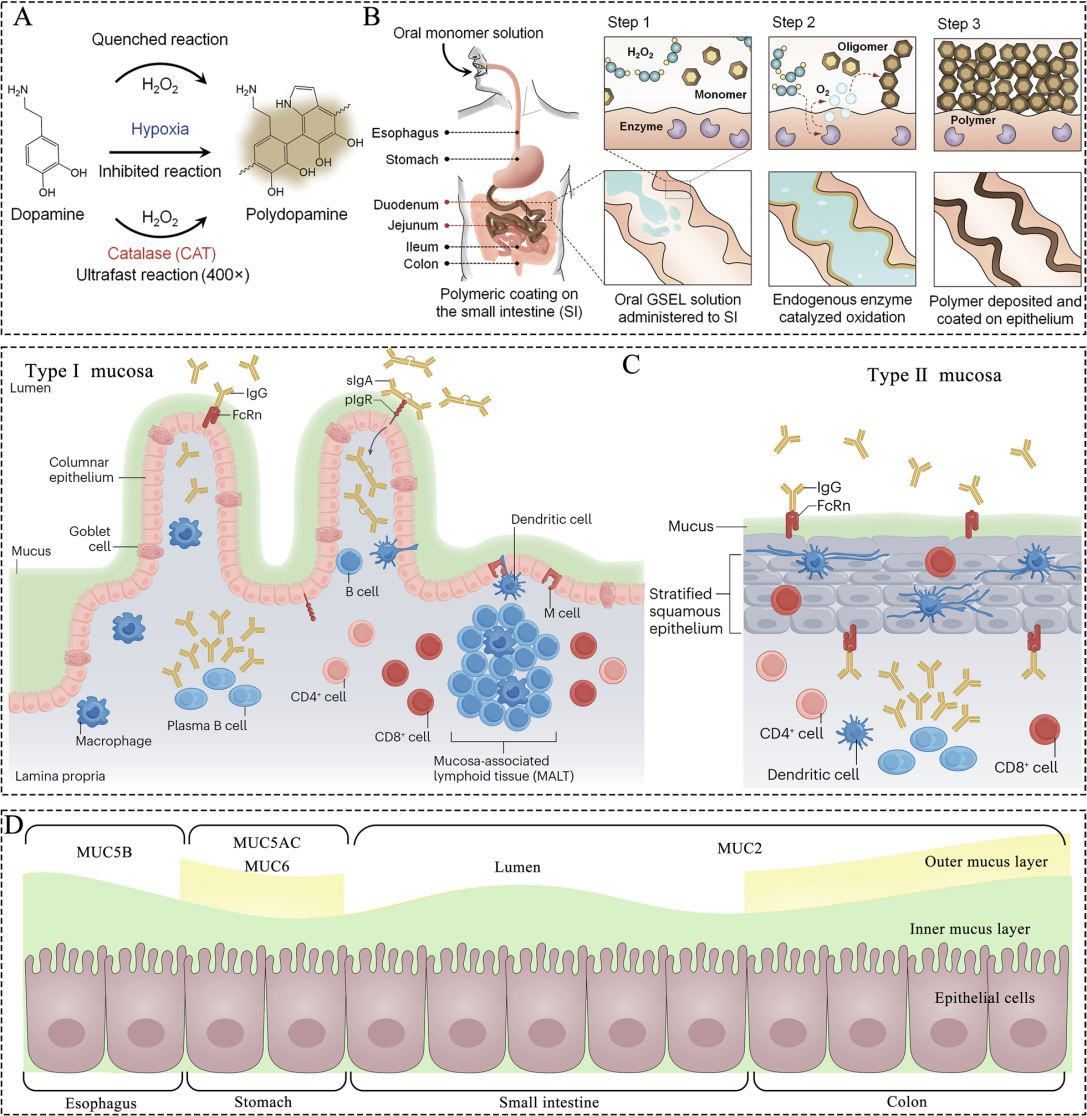
**A** Schematic illustration of catalase (CAT)-accelerated polydopamine (PDA) polymerization in a hypoxic environment. CAT catalyzes the conversion of hydrogen peroxide to oxygen, boosting oxygen release and accelerating PDA polymerization by approximately 400 times [57]; Reproduced with permission [57]. Copyright 2020, The American Association for the Advancement of Science. **B** Schematic illustration of the gastrointestinal synthetic epithelial lining (GSEL) technology. Orally administered DA monomers in the GSEL solution are rapidly oxidized in the presence of hydrogen peroxide, catalyzed by endogenous CAT. This results in the formation of a PDA coating on the small intestinal epithelium. Specific small intestine targeting and coating are facilitated by the uneven distribution of CAT along the GIT (with higher expression in the small intestine compared to the stomach and large intestine) [57]; Reproduced with permission [57]. Copyright 2020, The American Association for the Advancement of Science. **C** Mucosal tissues serve as the barrier between the human body and its surroundings and can be classified as type I and type II mucosa [62]; Reproduced with permission [62]. Copyright 2024, Springer Nature. **D** Schematic representation of mucus layers across the digestive tract. The mucus is divided into two layers: the outer mucus layer and the inner mucus layer. Both the stomach and colon have two layers, while the esophagus and small intestine feature a single mucus layer. Mucin is secreted differently in various GIT regions: MUC5B is found in the salivary glands and esophagus, MUC5AC and MUC6 in the stomach, and MUC2 in the small intestine [63, 64]

Most previous studies on small intestinal targeting focused on factors such as the pH differences between the small intestine and stomach, and the adhesion properties of the small intestine mucosa [55, 56]. Recently, a novel technique for targeting the small intestine, known as GSEL, has been developed [57]. This method is based on two key design concepts: first, to identify and apply endogenous enzyme-dependent reactions to control the efficiency of polymeric coating for specific anatomical targeting, and second, to develop tissue-specific catalytic polymerization techniques for the in-situ growth of building blocks on the intestinal epithelial surface [57, 58]. In this approach, polydopamine (PDA, a mussel-inspired tissue adhesive) is combined with catalase (CAT) to demonstrate its feasibility (Fig. 6A) [57]. The oral solution, containing dopamine (DA) monomers and trace amounts of hydrogen peroxide, induces the GSEL coating on the surface of the small intestine after passing through the mouth, esophagus, and stomach (Fig. 6B) [57]. Once in the small intestine, hydrogen peroxide molecules spread freely between the epithelial cells. Upon diffusion into or absorption by the intestinal epithelial cells, hydrogen peroxide is rapidly broken down by intracellular CAT into oxygen [58]. This oxygen interacts with extracellular DA monomers, which are oxidized into oligomers and polymers, forming a thin, strong PDA coating on the epithelial surface [59]. Because CAT expression is higher in the small intestine compared to other parts of the GIT, such as the stomach and colon, small intestinal targeting is achieved through the anatomical-specific distribution of natural enzymes along the digestive tract [60]. Building on the GSEL method, Li et al. synthesized a catechol-functionalized polymer by modifying alginate with DA [61]. The alginate-DA mixture, combined with other GSEL components, forms a gel on the surface of the small intestine after oral ingestion, due to the high CAT enzyme concentration in the small intestine. The oxidized catechol groups of DA covalently react with biomolecules on the small intestine, creating a firm adhesion in the hydrated intestinal environment. In a pig model, the pharmacokinetics of both hydrophobic levodopa and hydrophilic amoxicillin were demonstrated, indicating that this system could potentially deliver a wide range of drugs with different physicochemical properties [61]. Additional representative experiments for small intestinal targeting are summarized in Table 1.

## ODDSs for intestinal mucus targeting and penetration

Mucosal tissues serve as a barrier between the human body and its surroundings and can be classified into type I and type II mucosa (Fig. 6C) [62]. Type I mucosa lines the respiratory tract and GIT, while type II mucosa lines the mouth, esophagus, and cornea [65]. Although the morphology of each mucosal surface differs, all mucosal tissues share a common structure consisting of the mucus layer, epithelial layer, and lamina propria [63, 65]. The concentration of mucin in mucus (2–5%) determines its degree of crosslinking and viscoelasticity. Mucin is a glycoprotein, with its main protein chain composed of repeated serine, proline, and threonine residues, interspersed with hydrophobic, cysteine-rich domains. These domains contribute to polymerization, providing adhesion and swelling properties [66]. Additionally, mucins are heavily glycosylated with fucose, galactose, sialic acid, N-acetylgalactosamine, and N-acetylglucosamine to protect them from enzymatic hydrolysis in the digestive environment. This glycosylation helps restrict pathogen entry into the epithelial layer, acting as the first mucosal barrier [62]. However, the mucus layer varies across the GIT. In the stomach and colon, there is an inner, firmly adhered layer and an outer, loosely attached layer, while the small intestine contains only a single loosely attached mucus layer (Fig. 6D) [63, 66].

Given that mucins are negatively charged, one effective method for targeting adhesion is to construct negatively charged ODDSs. Chitosan, a commonly used positively charged material, serves as a building block for such systems. For instance, nanostructured lipid ODDSs loaded with curcumin (with a surface charge of − 34.02 mV) were prepared and coated with chitosan through EI. This modification resulted in a surface charge of + 32.13 mV for the delivery system [67]. Octenyl succinic anhydride-modified resistant starch was then applied as the outermost layer. In simulated intestinal fluid, the starch was digested, revealing the positively charged system. As a result, the curcumin-loaded ODDSs were significantly trapped in the colonic mucus, exhibiting a fluorescence intensity 18.35 times that of free curcumin [67]. EIs, such as these non-covalent bonds, remain a primary strategy for targeted delivery to the small intestine. However, recent studies have developed mucus-adhesive ODDSs through covalent interactions [68]. For example, Zhao et al. synthesized 4-phenoxynicotinamide derivatives of an intestine-targeted G-protein-coupled receptor 5 agonist, with side chains containing maleimides of varying chain lengths. Maleimides in this system could form covalent bonds with cysteine in mucin through a Michael addition reaction. After incubation with rat intestines for 2 h, the delivery system demonstrated excellent adhesion [68].

**Fig. 7:**
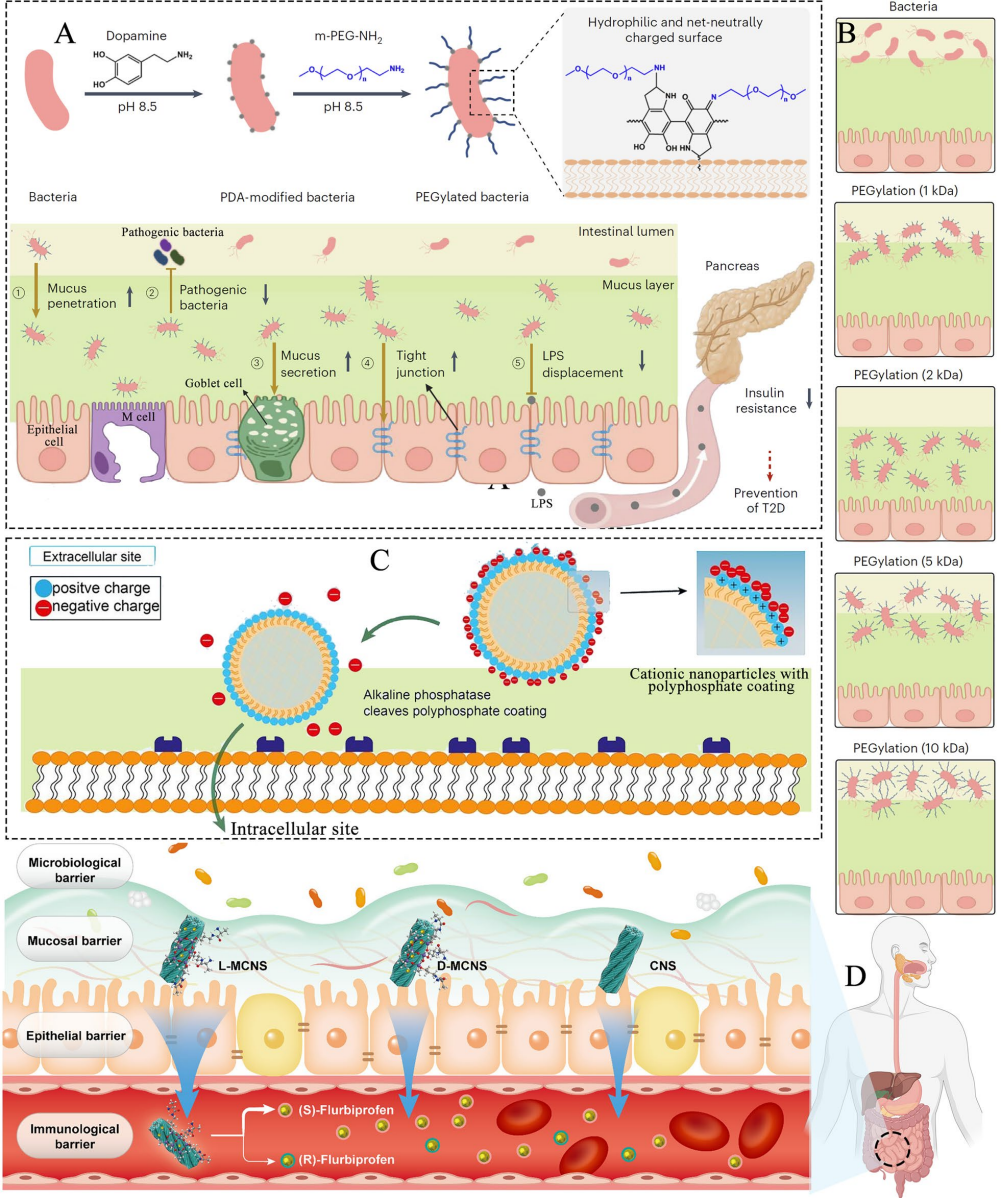
**A** Schematic illustration of the preparation of PEGylated bacteria through in situ self-polymerization of dopamine. PEGylation-mediated mucus penetration of bacteria can stimulate mucus secretion, enhance tight junction protein expression, and reinforce the gut mucosal barrier by preventing pathogenic bacterial invasion [70]; Reproduced with permission [70]. Copyright 2024, Springer Nature. **B** Bacteria grafted with PEG of varying chain lengths exhibit different mucosal penetration capabilities [70]; Reproduced with permission [70]. Copyright 2024, Springer Nature. **C** Negatively charged nanoparticles modified with phosphate substructures can easily traverse the mucus layer. Upon reaching the absorption membrane, the phosphate group is degraded, converting the system's charge to positive. This approach not only enables nanoliposomes to penetrate the mucosa but also significantly enhances their uptake by epithelial cells [71]; Reproduced with permission [71]. Copyright 2022, Elsevier. **D** Schematic illustration of the physiological barrier (including microbiological, mucosal, epithelial, and immunological barriers) and the primary processes involved in the oral absorption and blood distribution of flurbiprofen-loaded chiral silica nanoscrews and multichiral mesoporous silica nanoscrews [72]; Reproduced with permission [72]. Copyright 2024, American Chemical Society

Notably, while mucosal adhesion ODDSs exhibit excellent intestinal retention, their efficiency in penetrating the mucosa is limited, and they tend to be trapped in the mucus layer. Thus, such ODDSs are typically used for models of drug release within the intestine [69]. Nanoparticles with a negative to neutral surface charge are more likely to penetrate the mucus layer. For example, the use of poly(ethylene glycol) (PEG)-mediated bacteria has been proposed to enhance mucosal penetration (Fig. 7A) [70]. PEGylation creates a neutral and more hydrophilic surface, promoting the mobility and penetration of modified bacteria into the mucus. In the presence of DA, amine-terminated PEG (m-PEG-NH_2_) binds to PDA, allowing it to form and deposit in-situ on the surface of various bacteria under cytocompatible conditions. Surface modification with PEG of 2 kDa molecular weight provided the most efficient mucus penetration (Fig. 7B) [70].

However, ODDSs with a negative to neutral surface charge are not readily absorbed by epithelial cells, as epithelial cells tend to prefer positively charged particles. To improve both mucus penetration and cellular uptake, Charge-reversible ODDSs have been developed [69]. For instance, cationic ODDSs can be coated with anionic polymers based on methyl methacrylate and methacrylic acid to obtain a negatively charged surface delivery system (Fig. 7C) [71]. Negatively charged nanoparticles modified with phosphate substructures can easily pass through the mucus layer. Upon reaching the absorption membrane, the phosphate groups are decomposed, causing the system's charge to become positive. This not only facilitates mucus penetration but also enhances absorption by epithelial cells [71].

To further improve mucus penetration, ODDSs inspired by the spiral structures of bacteria and viruses have been developed. Inspired by the hierarchical chiral architecture of *Spirillum*, Yumei Wang et al. prepared multichiral mesoporous silica nanoscrews with a chiral helix shape using chiral templates and post-modification strategies [72]. The helical structure presented a unique topological form, with a large rough exterior, small cross-sectional area, and a screw-like body. During drug administration, these nanoscrews demonstrated multiple advantages in mucosal diffusion and adhesion (Fig. 7D) [72]. Similarly, inspired by viral structures, Bao et al. used amphiphilic peptides obtained via enzymatic hydrolysis to self-assemble, forming long nanotubes, short nanotubes, cross-linked short nanotubes, large nanospheres, and small nanospheres [73]. Among these, the short nanotubes mimicked artificial viruses, penetrating the mucus and demonstrating high bioavailability for oral administration [73]. Additional representative experiments for mucus targeting are summarized in Table 1.

## The transepithelial transport of ODDSs

### Paracellular transport and enhancement strategies

**Fig. 8:**
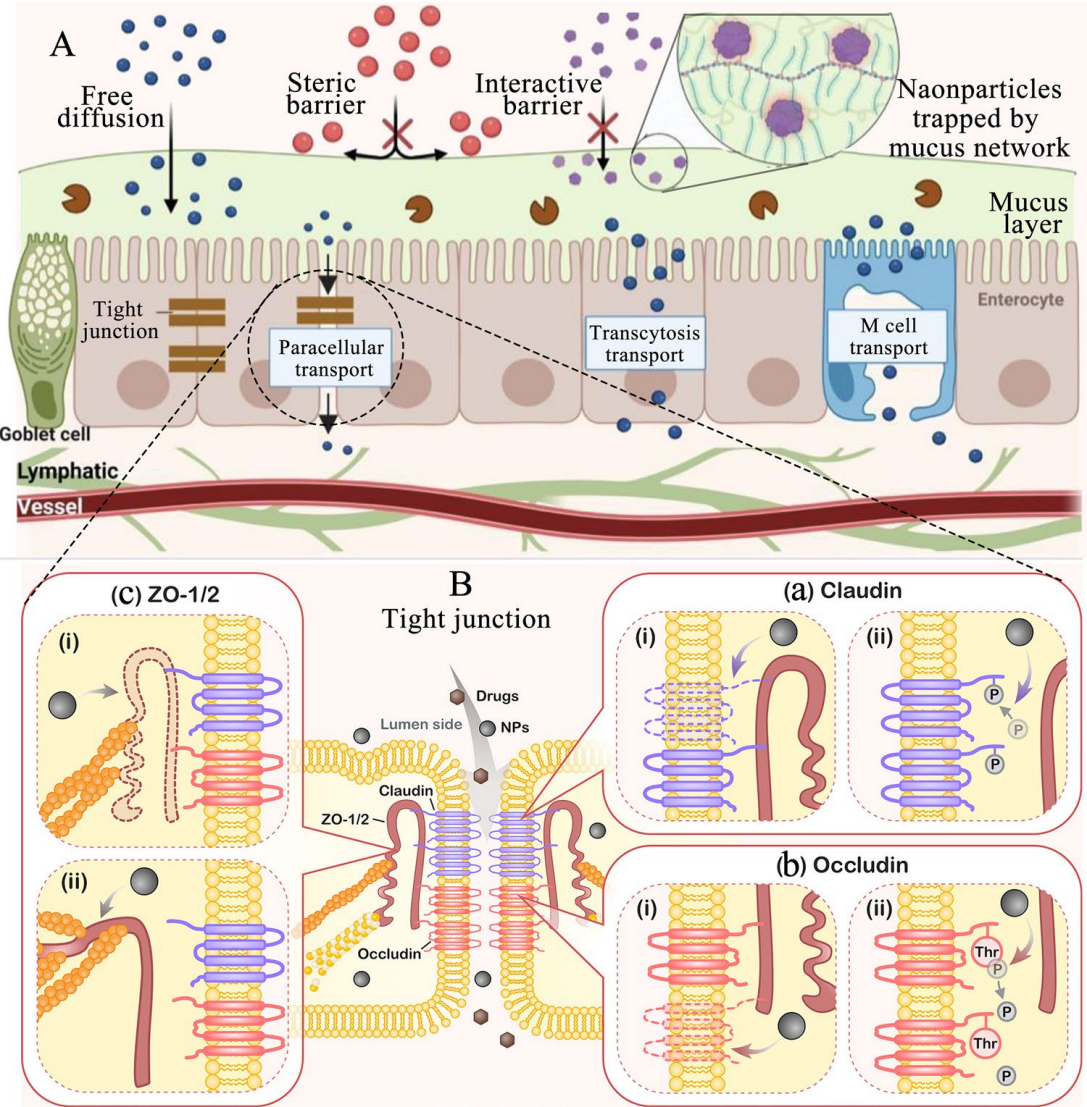
**A** Schematic diagram of transepithelial transport in the GIT for ODDS, including paracellular transport, transcytosis pathway, and microfold cells (M cells)-associated pathway [74]. **B** Mechanism for opening tight junctions through direct interaction with tight junction proteins (including claudin, occludin, and ZO-1/2) [81]. Reproduced with permission [81]. Copyright 2022, American Chemical Society

Paracellular transport refers to the movement of ODDSs or drugs into the bloodstream through the spaces between intestinal epithelial cells, rather than directly through the epithelial monolayers, as illustrated in Fig. 8A [74]. Additionally, Fig. 8A shows that only ODDSs that can freely diffuse through the mucus layer have the opportunity to enter the bloodstream via the transepithelial transport pathway. Both steric and interactive barriers prevent ODDSs from making contact with the epithelial cells. Beyond paracellular transport, other mechanisms such as transcytosis and M cell-associated pathways have also been identified [74] (Fig. 8A). Paracellular transport is primarily limited by tight junctions (TJs) between intestinal epithelial cells. These junctions act as a barrier to the invasion of pathogens and exogenous substances. TJs are composed of proteins such as claudins, zonula occludens-1 (ZO-1), occludins, and junctional adhesion molecules, which form a dynamic barrier regulated by epithelial cells [75]. There is selective permeability between the basolateral and apical membrane domains [76]. ZO-1 interacts with the cytoskeleton protein F-actin and transmembrane proteins, playing a key role in the formation of TJs by mediating mechanical perception through TJ turnover and protein phase separation [77]. The expression of TJ proteins varies along the GIT, and these differences may be linked to the varying exposure of intestinal segments to lumen substances [78]. TJs serve three main functions: first, they provide adhesion and maintain the polarity and integrity of epithelial tissue; second, they control the selective passage of water, ions, molecules, nutrients, and pathogens through the epithelium [79]; and third, they participate in signal transduction, receiving and transmitting signals that influence tissue function and cell behavior, including apoptosis and differentiation [78]. Thus, TJs provide mechanical strength and regulate the permeability of the epithelial barrier by altering the abundance or composition of proteins. When fully opened, the gap between cells is approximately 20 nm. Therefore, nanoparticles with diameters greater than 12 nm face significant challenges in passing through TJs [74]. In intact intestinal mucosa, the amount of nanoparticles transported via this pathway is typically less than 10%, depending on factors such as particle size and the integrity of the epithelial junctions [80]. Consequently, achieving effective paracellular transport remains a significant obstacle for ODDSs.

Paracellular transport is a passive process that requires the opening of TJs between cells for ODDSs to pass through this pathway. A key mechanism for opening TJs involves direct interaction with the TJ proteins (Fig. 8B) [81]. As shown in Fig. 8B, ODDSs can influence claudins by (i) decreasing the protein levels of specific claudins, or (ii) modulating claudin phosphorylation, both of which can alter the affinity of claudins for ZO proteins. Additionally, ODDSs may affect occludin by (i) reducing its protein level or (ii) by lowering the phosphorylation level of its threonine/serine sites. Similarly, ODDSs can impact ZO-1 by (i) reducing its mRNA and protein levels or (ii) by altering its conformation, either folding or stretching (Fig. 8B) [81]. Claudins are considered the most critical paracellular proteins, as they are essential for forming TJs. The C-terminal cytoplasmic portion of claudins directly binds to cytoplasmic scaffold proteins [82]. ODDSs can influence claudins by reducing the protein level of specific claudins or by modulating their phosphorylation, which in turn changes their affinity for ZO proteins [79]. For example, chitosan-based ODDSs, known for their good biocompatibility, can reduce claudin levels and reversibly open TJs [81]. Similarly, ODDSs can affect occludin by reducing its protein level or by decreasing the phosphorylation of its threonine or serine sites [79]. For ZO-1, ODDSs can reduce its mRNA levels or regulate its conformational changes to influence ZO-1 function [83]. Several substances are known to reversibly open TJs. Surfactants can disrupt TJs by binding to charged surface proteins, denaturing them. Common anionic surfactants include sodium dodecyl sulfate (SDS), while cationic surfactants like benzalkonium chloride (BZC) and sodium dodecyl benzene sulfonate (SDBS) are also frequently used [84]. An ODDS loaded with insulin was prepared using 1,2-diolyl-3-trimethylammonium propane as cationic liposomes, and the results demonstrated that it could open TJs and promote insulin paracellular transport [85]. Substances that chelate bivalent cations, such as calcium or magnesium ions, also facilitate TJ opening; examples include phytic acid, citrate, and ethylene diamine tetraacetic acid [86]. Sodium caprate is another potent penetrant that can reversibly regulate TJs via calmodulin-dependent contraction of actin-myosin filaments, induced by phospholipase [87]. Additionally, other substances such as cell-penetrating peptides and chitosan have been reported to reversibly open TJs [88]. Incorporating these substances into ODDSs provides an effective strategy to enhance paracellular transport.

While opening TJs, a natural barrier, can enhance ODDS delivery, it also carries potential risks. Although TJ opening facilitates efficient ODDS transport, it may allow dietary antigens or pathogens (such as viruses, bacteria, and lipopolysaccharides) to cross the barrier irreversibly, alongside the ODDSs [89]. These antigens or pathogens are often present in the intestinal tract either permanently or opportunistically. Increased intestinal permeability has been linked to a higher incidence of autoimmune diseases, including infections and inflammatory bowel disease (IBD) [78, 90]. Furthermore, heightened intestinal permeability may increase the likelihood of developing type I diabetes and the propagation of graft-*versus*-host disease [91]. Opening TJs with ODDSs may be transient, with recovery occurring within a few hours or days. However, it remains unclear whether periodic, repeated openings of TJs will surpass the body's repair capacity [92]. A notable example is sodium caprate, a well-known TJ opener used in GIPET^®^ technology [93]. While sodium caprate has been approved as a food additive in the United States and the European Union and is used in the phase II clinical trial of oral insulin delivery by Novo Nordisk, the company eventually halted the oral insulin delivery plan. Despite the clinical success, the decision was made partly due to low oral absorption efficiency, but also because of the potential cross-cellular interference caused by the penetrant [93]. Additionally, ODDSs that can open TJs should be used with caution in patients with IBD or celiac disease, as their TJs may already be compromised to some extent [89]. Even for ODDSs constructed from natural substances—typically considered safe—the risks of opening TJs cannot be overlooked. For example, although bile acids are naturally secreted compounds in the body, excessive bile acids can disrupt the structure and barrier function of TJs [94]. Similarly, a high-carbohydrate, fast-food diet may contribute to the development of type II diabetes, which is associated with increased intestinal permeability and protease repositioning. A carbohydrate-rich diet can alter the expression of occludin and ZO-1 proteins, further increasing intestinal permeability [95, 96]. Many ODDSs are often formulated with carbohydrate-based shells, which underscores the importance of ensuring their safety before use.

### Transcytosis pathway and targeting strategies

**Fig. 9:**
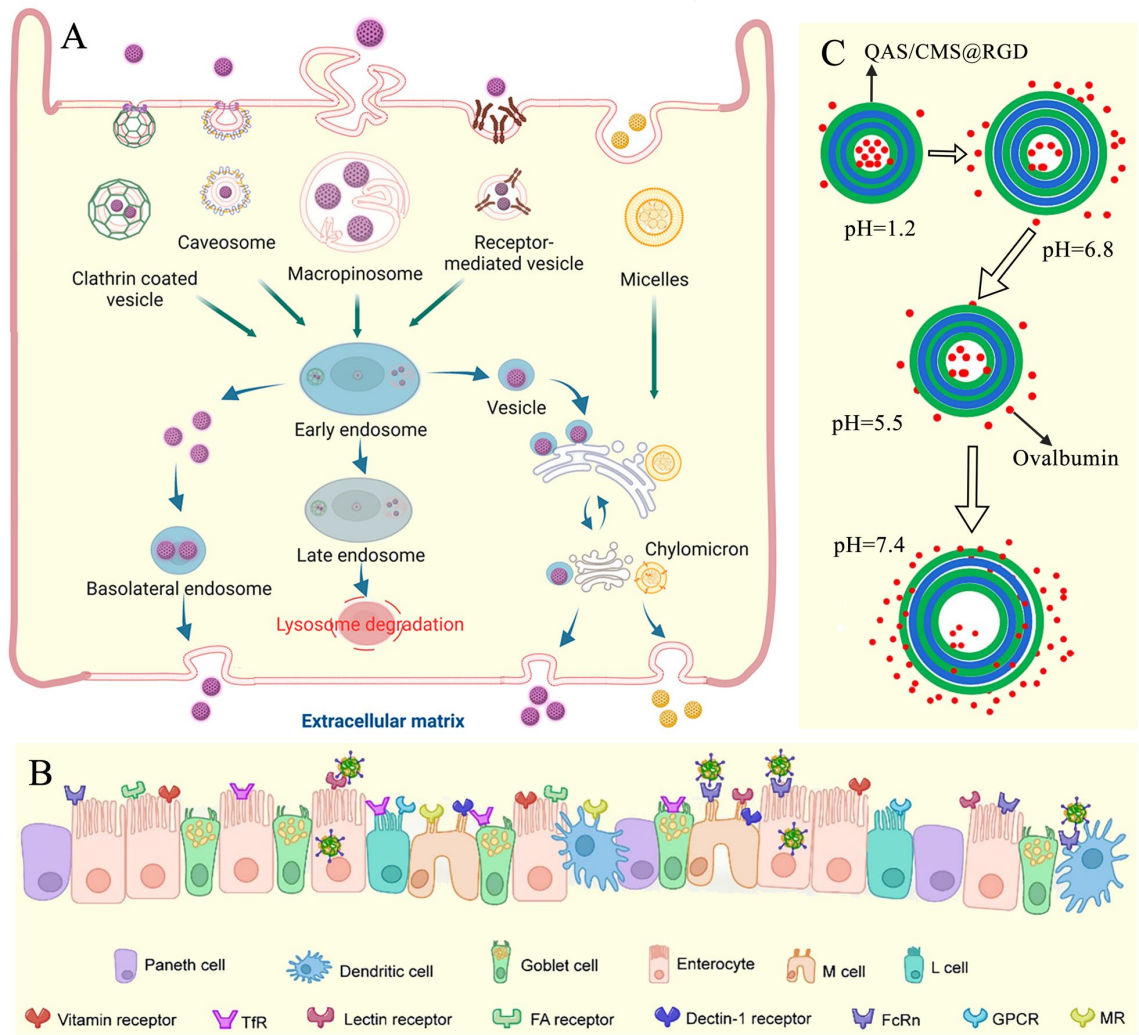
**A** Schematic diagram of the possible endocytic pathways through which particles are transported by intestinal epithelial cells, including clathrin-, caveolin-, micropinocytosis-, and receptor-mediated endocytosis [98]; Adapted with permission [98]. Copyright 2023, American Chemical Society. **B** Endocytosis process and receptor targeting via clathrin-mediated absorption, highlighting receptors for vitamins, transferrin (TfR), lectins, follicle-associated (FA) cells, dectin-1, folate (Fc), and G protein-coupled receptors (GPC) across different intestinal epithelial cells, including Paneth cells, dendritic cells, goblet cells, enterocytes, M cells, and L cells [109]; Adapted with permission [109]. Copyright 2022, Elsevier. **C** Effect of pH changes (simulated gastric fluid: pH 1.2, simulated intestinal fluid: pH 6.8, simulated cellular fluid: pH 5.5, and simulated tissue fluid: pH 7.4) on the electrostatic interaction (EI) of building blocks in nano-capsules. The increase in pH results in a decrease in affinity constants, facilitating successful ovalbumin delivery to M cells [132]; Adapted with permission [132]. Copyright 2024, Elsevier

The transcytosis pathway serves as the primary route for ODDSs to enter systemic circulation (Fig. 8A) [74]. ODDSs typically undergo a series of processes as they traverse the intestinal epithelial cell layer, including apical endocytosis, intracellular trafficking, and basolateral exocytosis [97]. Initially, ODDSs enter epithelial cells from the apical surface through clathrin-mediated endocytosis, macropinocytosis, or caveolae-mediated endocytosis (Fig. 9A) [98]. As shown in Fig. 9A, ODDSs encounter various compartments and organelles within the cell. They may pass through early and late endosomes, eventually reaching lysosomes where degradation may occur. Alternatively, ODDSs can follow the endoplasmic reticulum/Golgi complex pathway and be released extracellularly (Fig. 9A) [98]. Clathrin is a triskelion protein that plays essential roles in different cellular processes, including endocytosis, mitosis, and cell migration [98]. Clathrin-mediated endocytosis is the primary mechanism for receptor-mediated uptake of substances like antibodies, hormones, and nutrients [99]. This process involves vesicle formation, transport to intracellular destinations, and potential exocytosis [98]. Macropinocytosis, a process considered evolutionarily conserved and independent of clathrin or vesicle-mediated pathways, promotes the non-specific uptake of extracellular fluid and its contents. It also contributes to the internalization of apoptotic cell debris and viruses or bacteria, either intact or fragmented [100]. Macropinocytosis is unique in its ability to engulf larger particles (200–1000 nm), even reaching the micron scale [98]. It occurs in almost any cell and serves as an alternative pathway in cells that cannot perform phagocytosis [98, 101]. Earlier studies suggest that positively charged drug delivery systems are primarily internalized through micropinocytosis [102]. Caveolae, which are flask-shaped invaginations formed from plasma membrane lipid raft domains, are involved in various cell functions, including signaling, membrane protection, cholesterol homeostasis, and endocytosis [103]. Caveolae-mediated endocytosis follows a complex process involving caveolae formation, assembly, shedding, and intracellular transport [104]. Key endocytosis proteins, such as caveolins, along with cavins, adaptor proteins, and stabilizing molecules, provide structural scaffolds for the process [104]. ODDSs entering via caveolae-mediated endocytosis are localized in caveosomes, which are then absorbed by the endoplasmic reticulum and Golgi apparatus for delivery or exocytosis, bypassing lysosomal hydrolysis [105]. Anionic ODDSs are often endocytosed via the caveolae-mediated pathway [103]. Once ingested, ODDSs undergo complex intracellular transport within endosomes or transport vesicles (Fig. 8A) [74]. The transport process within cells typically follows the degradation pathway, secretion pathway, and recycling pathway [106]. The degradation pathway involves the transport of nanoparticles through the endosome-lysosome route [106], where enzymes and the acidic lysosomal environment (pH 4.5–5.5) degrade proteins and lipids of the delivery systems, significantly reducing transport efficiency [107]. In contrast, transport via secretory and recycling pathways can bypass lysosomal degradation, preserving the structural integrity of ODDSs and enhancing their cellular transport [106, 107].

To enhance the targeting of intestinal epithelial cells and improve transcytosis absorption, a fundamental strategy involves incorporating ligands onto the surface of ODDSs. Ligands not only facilitate cellular targeting but also improve pharmacokinetics, reduce toxicity to healthy tissues, enable personalized medicine, and minimize drug resistance [108]. In the process of enhancing endocytosis, many receptors primarily utilize clathrin-mediated uptake. Notable receptors include transferrin, vitamin receptors, and carbohydrate receptors (Fig. 9B) [109, 110]. The transferrin receptor is a homodimeric membrane receptor (90 kDa per subunit) responsible for the active absorption of transferrin. Following transferrin's endocytosis, the ligand-receptor complex undergoes recycling, releasing intracellular transferrin-bound iron [110]. In a study by Zhou et al., PEG-poly(lactic-co-glycolic acid) nanoparticles loaded with Notoginsenoside R1 were modified with transferrin [111]. The study found that when the delivery system was not modified with transferrin, the particles entered cells via caveolae-mediated endocytosis. However, transferrin-modified systems were internalized through clathrin-mediated endocytosis. In rat intestines, the enrichment of transferrin-modified nanoparticles in the colon was significantly higher compared to unmodified particles [111]. Several vitamins also bind specifically to receptors on intestinal epithelial cells, such as vitamin B_12_ and folic acid [112].

Vitamin B_12_, a cobalt-containing vitamin synthesized by microorganisms, forms a complex with intrinsic factor, which is recognized by specific epithelial receptors and internalized via clathrin-mediated endocytosis, facilitating transport across intestinal cells [113]. This property has been utilized to enhance ODDS absorption by chemically coupling vitamin B_12_ to nanocarriers. Folic acid, essential for cell division [114], binds to a variety of genetically distinct folate receptors in the gut, including the proton-coupled folate transporter (PCFT), reduced folate carrier (RFC), and folate receptors FRα and FRβ [115]. These receptors promote rapid endocytosis and efficient transmucosal transport, similar to the mechanism used by the neonatal fragment crystallizable receptor that transports bound macromolecules into RAB11- and RAB4-positive apical recycling endosomes [116]. For example, drugs delivered via folic acid-modified liposomes are absorbed through metabolically sensitive root inhibitor pathways and pH-dependent pathways [117]. Moreover, intestinal epithelial cells, including dendritic cells, goblet cells, and Paneth cells, express various carbohydrate receptors, such as mannose and galactose receptors. The mannose receptor family includes four endocytic protein receptors: DEC-205/CD205/gp200-MR6, Endo180/uPARAP, the mannose receptor, and the M-type receptor for phospholipases A2 [118]. These receptors are highly expressed in dendritic cells and play pivotal roles in immune responses. Therefore, ODDSs with mannose modification targeting the intestinal tract can promote transcytosis [119]. Galactose also functions as a targeted ligand, binding to sodium-glucose cotransporters and recognizing glucose transporter 2 during transient insertion into the apical membrane. Receptors for galactose are expressed on dendritic and endocrine cells within the intestinal tract [113].

### Microfold cells (M cells)-associated pathway and targeting strategies

M cells, primarily located in Peyer's patches, are specialized epithelial cells characterized by membrane or basement membrane invagination and are integral to the mucosal immune system (Fig. 8A) [74]. In contrast to columnar enterocytes, M cells have underdeveloped glycocalyx and microvilli structures, with lower levels of membrane hydrolase activity. Additionally, the follicle-associated epithelium contains few goblet cells, resulting in minimal or no mucus, thereby facilitating the efficient uptake of ODDSs via M cells [120]. The unique architecture of M cells enables the selective transport of specific substances into the underlying lymphoid tissue through active transport mechanisms, offering a potential strategy to enhance ODDS bioavailability by bypassing first-pass metabolism and minimizing toxicity [121]. Despite comprising less than 1% of the total absorptive intestinal epithelium, M cells have been widely studied as targets for ODDS delivery [122]. M cells preferentially internalize ODDSs with diameters smaller than 10 μm, with particles ranging from 550 to 1100 nm demonstrating optimal absorption at Peyer's patches. This pathway accounts for approximately 0.2 to 70% of ODDS delivery efficiency, depending on factors such as size and surface area [74]. M-cell-specific targeting offers a means to overcome the limitations posed by the low number of M cells in oral drug delivery. A key strategy for targeting M cells involves conjugating ODDSs with suitable M-cell-targeting molecules, including lectins, RGD (Arg-Gly-Asp) peptides, and specific receptors.

#### Lectins

Lectins are natural proteins found in plants or bacteria that specifically bind to carbohydrate moieties on proteins or lipids [123]. These proteins are among the most extensively studied ligands for targeting M cells. Plant lectins can recognize specific carbohydrate residues present in the glycocalyx of the follicle-associated epithelium, making them useful in the development of M-cell-targeted ODDSs [124]. One of the most widely used lectins for targeting M cells is *Ulex europaeus agglutinin-1* (UEA-1), a fucose-specific lectin that binds to glycoproteins with a fucα1-2Galβ core located on the luminal side of M cells. Upon binding to ODDS, UEA-1 facilitates their absorption by M cells when taken orally [125]. Tomato lectin, which is specific for N-acetylglucosamine, enhances the adhesion of ODDSs to intestinal epithelial cells [126]. For example, when tomato lectin was covalently bound to the surface of 500 nm polystyrene particles, it not only influenced overall absorption but also increased the absorption rate by over ten times compared to unmodified particles. Furthermore, this modification significantly shifted the absorption site from lymphoid tissue to non-lymphoid intestinal tissue [76]. Wheat germ agglutinin (WGA) selectively binds to N-acetylglucosamine and sialic acid on the surface of M cells [127]. Hou et al. encapsulated shikonin and gefitinib in micelles coated with WGA. The presence of WGA significantly improved the mucosal penetration ability of the micelles, increased the uptake efficiency by intestinal cells, enhanced the oral bioavailability of shikonin and gefitinib, and overcame drug resistance, demonstrating promising anti-tumor activity [127]. While the binding of lectins to specific carbohydrate components in the mucus layer may limit their binding to epithelial cells, this interaction also enhances the mucosal adhesion of lectin-modified ODDSs, thereby prolonging their retention time. Furthermore, studies have shown that the rapid turnover of the mucous layer does not hinder the ability of lectins to target M cells effectively [128].

#### RGD (Arg-Gly-Asp) peptide

RGD (Arg-Gly-Asp) peptide is a tripeptide composed of the amino acids arginine (R), glycine (G), and aspartic acid (D) [129]. It is a cell adhesion motif found in various extracellular matrix proteins, including vitronectin, fibronectin, and laminin [129]. RGD plays a pivotal role in mediating cell migration, attachment, and differentiation by binding to integrin receptors on the cell surface [130]. Specifically, RGD recognizes α5β1 integrins on the apical surface of M cells. Through functional groups such as amine (–NH_2_), carboxylate (–COOH), and alcohol (–OH), RGD can form chemical bonds with ODDSs [129]. Modifying ODDSs with RGD offers several advantages. First, M-cell phagocytosis is a rapid process, typically occurring within 10–15 min. Second, RGD-modified ODDSs can bypass the surface charge requirements of the intestinal mucus layer. Third, this modification avoids the need for mucus dissolution or adhesion systems, which could compromise the integrity of the mucus and increase the risk of infection [131]. For example, Xie et al. grafted carboxymethyl starch (CMS) with RGD peptide and cationic quaternary ammonium starch to fabricate quintet-layered nano-capsules loaded with ovalbumin [132]. Changes in pH primarily affected the EI between the building blocks, with increased pH leading to decreased affinity constants, as shown in Fig. 9C [132]. Approximately 84.42% of the ovalbumin was successfully delivered to M cells. Furthermore, the delivery system was five times more efficient at transporting M cells compared to Caco-2 cells [132]. Similarly, Yu et al. used RGD peptide to modify doxorubicin-loaded solid lipid nanoparticles (DOX-SLNs). After oral administration in rats, the area under the plasma concentration–time curve of DOX increased by 2.25 times [131].

#### Receptor-mediated targeting

M cells play a critical role in the presentation and processing of various pathogens and antibodies. Consequently, receptors involved in these interactions serve as potential targets for M cell-specific delivery. Glycoprotein 2 (GP2), a highly expressed transcytotic receptor on the luminal membrane of M cells, is one such target [133]. GP2 is involved in mucosal barrier maintenance, immune system homeostasis, and antigen-specific immune responses [134]. It specifically recognizes type 1 fimbrial adhesin (FimH), a component of type 1 pili found in several pathogenic microorganisms, including *Salmonella typhimurium* and *Escherichia coli* [133]. When M cells are anchored, GP2 binds to bacteria, which are then internalized through trans-endocytosis. This process allows phagocytosis by underlying dendritic cells, initiating antigen-specific mucosal immune responses [135]. Shima et al. developed an effective antigen-delivery system using a fusion protein, anti-GP2-streptavidin [136]. Immunofluorescence studies confirmed that anti-GP2-streptavidin specifically bound to M cells [136]. Compared to biotinylated ovalbumin peptide alone or biotinylated ovalbumin peptide conjugated with streptavidin, orally administered biotinylated ovalbumin peptide (bOVA) conjugated with anti-GP2-streptavidin led to a significantly higher OVA-specific induction of fecal IgA secretion. This demonstrated that anti-GP2-streptavidin was an effective mucosal vaccine capable of withstanding the harsh gastrointestinal environment while efficiently delivering cargo to M cells [136]. Other potential targets for M-cell-specific delivery include Toll-like receptor-2 (TLR-2), Toll-like receptor-4 (TLR-4), α5β1 integrin, and the platelet-activating factor receptor [137]. These receptors interact with pathogen-associated molecular patterns (PAMPs), which help bacteria metastasize within the intestinal lumen. PAMPs such as peptidoglycan, lipoteichoic acid, lipopolysaccharides, bacterial flagellin, and CpG DNA are recognized by pathogen recognition receptors, promoting M-cell-mediated transport [137].

## Colon

**Fig. 10:**
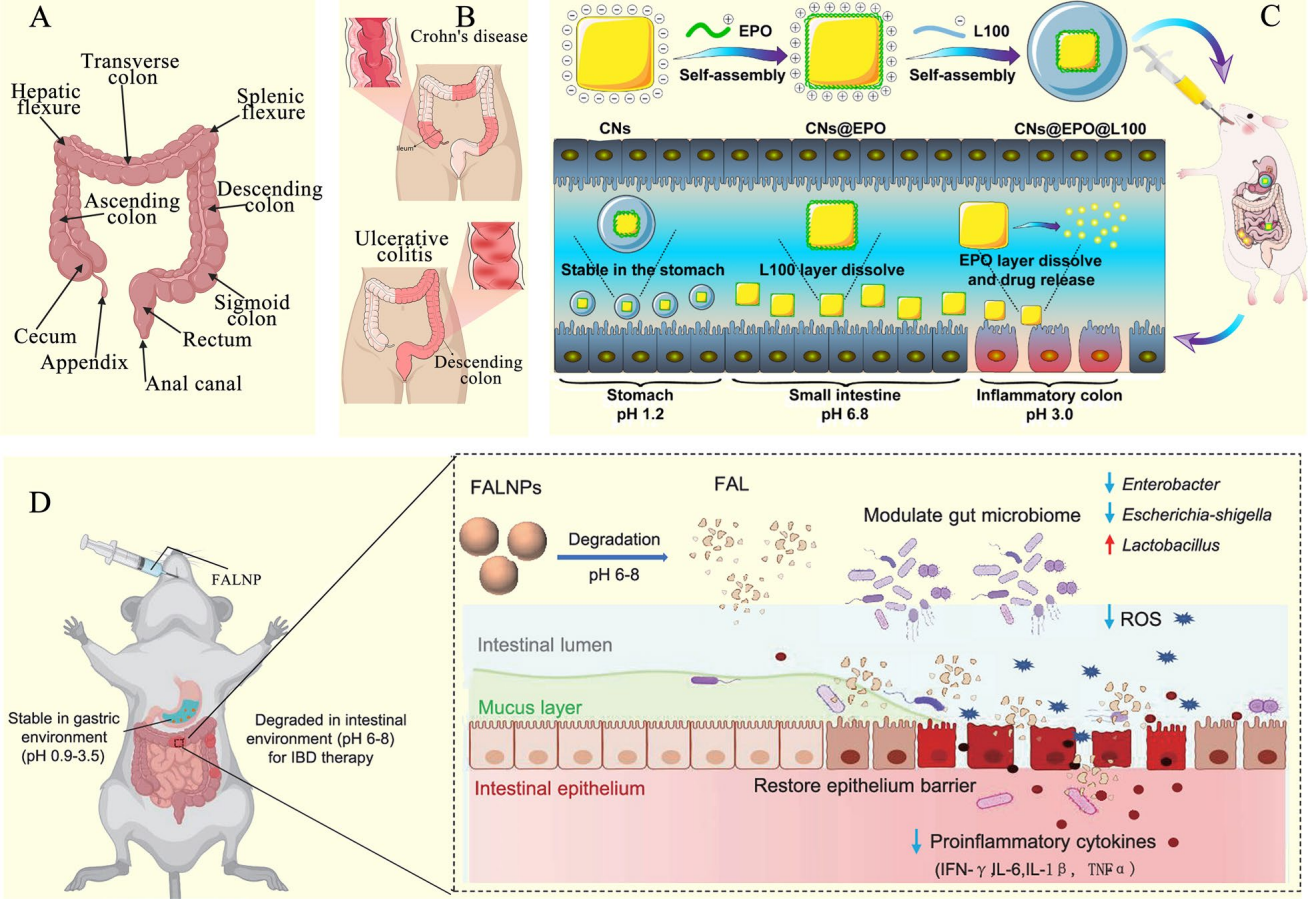
**A** The large intestine consists of an M-shaped tube, including the cecum, ascending colon, transverse colon, descending colon, sigmoid colon, and rectum, among other sections. **B** Ulcerative colitis (UC) extends from the rectum to the colon, affecting only the innermost mucosa, while Crohn’s disease (CD) can cause transmural inflammation throughout the gut [141]; Adapted with permission [141]. Copyright 2021, Elsevier. **C** Core–shell nanoparticles, prepared by coating nano-curcumin with Eudragit^®^ EPO and Eudragit^®^ L100, are pH-responsive for UC treatment. These nanoparticles protect nano-curcumin in the stomach and small intestine due to the Eudragit^®^ layers, ensuring enhanced availability at the UC site after oral administration [147]; Adapted with permission [147]. Copyright 2023, Royal Society of Chemistry (RSC). **D** FALNP, an orally administered therapeutic nano-reagent, treats IBD. It withstands the gastric environment and degrades in the intestinal milieu, producing ferulic acid-derived synthetic lignin polymers that alleviate intestinal inflammation by reducing reactive oxygen species, modulating gut bacteria, and regulating inflammatory cytokines [148]; Adapted with permission [148]. Copyright 2024, American Chemical Society

The large intestine is shorter than the small intestine, measuring approximately 1.5 m in length, but has a larger diameter (5–7 cm). It consists of an M-shaped tube, comprising the cecum (6–7 cm), appendix, ascending colon (15–20 cm), transverse colon (45–50 cm), descending colon (25–30 cm), sigmoid colon (~ 40 cm), rectum (~ 12 cm), canal, and anus (Fig. 10A) [138]. Though anatomically similar to the small intestine, the large intestine exhibits three key differences. First, it facilitates the transportation of digested food from the colon to the anus through peristalsis and retrograde movement of the proximal large intestine in response to gravity or reflux waves. Second, epithelial cells form folds with small crypts instead of villi, and the number of functional cells is reduced. Third, the large intestine houses a significant population of microbes [139, 140]. These distinctions influence the design of targeted delivery strategies for the large and small intestines. The colon, being the primary section of the large intestine, is more susceptible to diseases such as IBD, colon cancer, and colonic dyskinesia [17]. IBD is a group of autoimmune disorders characterized by chronic inflammation of the GIT, with unclear pathogenesis. The two main types of IBD, ulcerative colitis (UC) and Crohn’s disease (CD), differ primarily in the location of inflammation within the GIT, as illustrated in Fig. 10B [141]. UC affects the colon and rectum, involving only the innermost mucosa, while CD is characterized by transmural inflammation that can affect the entire gut [141]. This section focuses on the use of ODDSs for colonic targeting and IBD treatment. In conclusion, ODDSs designed for colonic targeting leverage several factors unique to the colon, including its more alkaline pH, the presence of intestinal flora, elevated reactive oxygen species (ROS), and distinct charge conditions in diseased regions.

### pH-dependent target-action response mode

In the digestive tract, the pH is the lowest in the stomach, ranging from 1.0 to 2.5, while the proximal small intestine exhibits a pH range of 6.15 to 7.35, and the distal region can reach up to 7.5. The colon's pH ranges from 5.26 to 6.72, with non-digestible dietary carbohydrates being converted by colonic bacteria into short-chain fatty acids, leading to a reduction in colon pH [6, 142]. These significant pH variations across the digestive tract can be leveraged to design pH-responsive ODDSs that protect drugs from stomach acids and enzymes. Once the ODDS reaches the intestine, it must effectively dissolve or expand in the neutral pH environment, ensuring targeted drug release in the colon [143]. The most common pH-responsive mechanism involves the alternating protonation and deprotonation of COOH-COO^–^ groups in the polymer building blocks [144]. As the ODDS passes through the stomach, the carboxyl group becomes protonated at the low pH, effectively protecting the drug from stomach acid and enzymes. Upon reaching the intestine, the carboxyl group is deprotonated due to the higher pH, causing electrostatic repulsion and swelling, which facilitates the release of the drug from the ODDS [144]. Commonly used building blocks include anionic polymers, acrylic acid esters, and methacrylic acid copolymers, such as Eudragit [143, 145]. Eudragit can be tailored with various side chains, producing derivatives responsive to different pH gradients. Generally, the more carboxylic acid groups in the polymer, the lower its pH threshold and the faster it dissolves under neutral conditions (and vice versa) [146]. For instance, Eudragit L100 dissolves at pH 6, while Eudragit S100 dissolves at pH 7, and the two can be combined in different ratios to control the pH at which the ODDS dissolves and releases. Eudragit is FDA-approved for use in oral formulations [145]. In a study by G. Zhang et al., curcumin was encapsulated in Eudragit^®^ EPO and L100 to form core–shell nanoparticles (Fig. 10C) [147]. This system demonstrated programmed pH-dependent release and enhanced the anti-inflammatory effects of curcumin, addressing its poor dissolution in the stomach and small intestine, and enabling targeted therapy for UC [147]. In addition to synthetic pH-responsive materials, natural product-derived pH-responsive particles have been developed, such as ferulic acid-derived lignin nanoparticles (FALNP) [148]. FALNP exhibits intestinal pH-responsive degradability, allowing it to withstand the harsh acidic conditions of the stomach, bypass physiological barriers, and specifically target the intestinal tract. Oral administration of FALNP can significantly alleviate the pathological symptoms of IBD by reducing oxidative stress and modulating the intestinal microbiota (Fig. 10D) [148]. However, although pH-dependent ODDSs have been successfully developed, they primarily target the entire colon rather than specific inflammatory regions. This broad targeting may result in insufficient accumulation in the tissues of inflamed colonic lesions, potentially leading to adverse reactions. Furthermore, disease conditions can alter colon pH. For instance, patients with UC exhibit a colon pH of approximately 3.0 [142]. Consequently, single pH-responsive ODDSs have certain limitations in effectively addressing localized inflammation.

### Colonic microbial-dependent target-action response mode

**Fig. 11:**
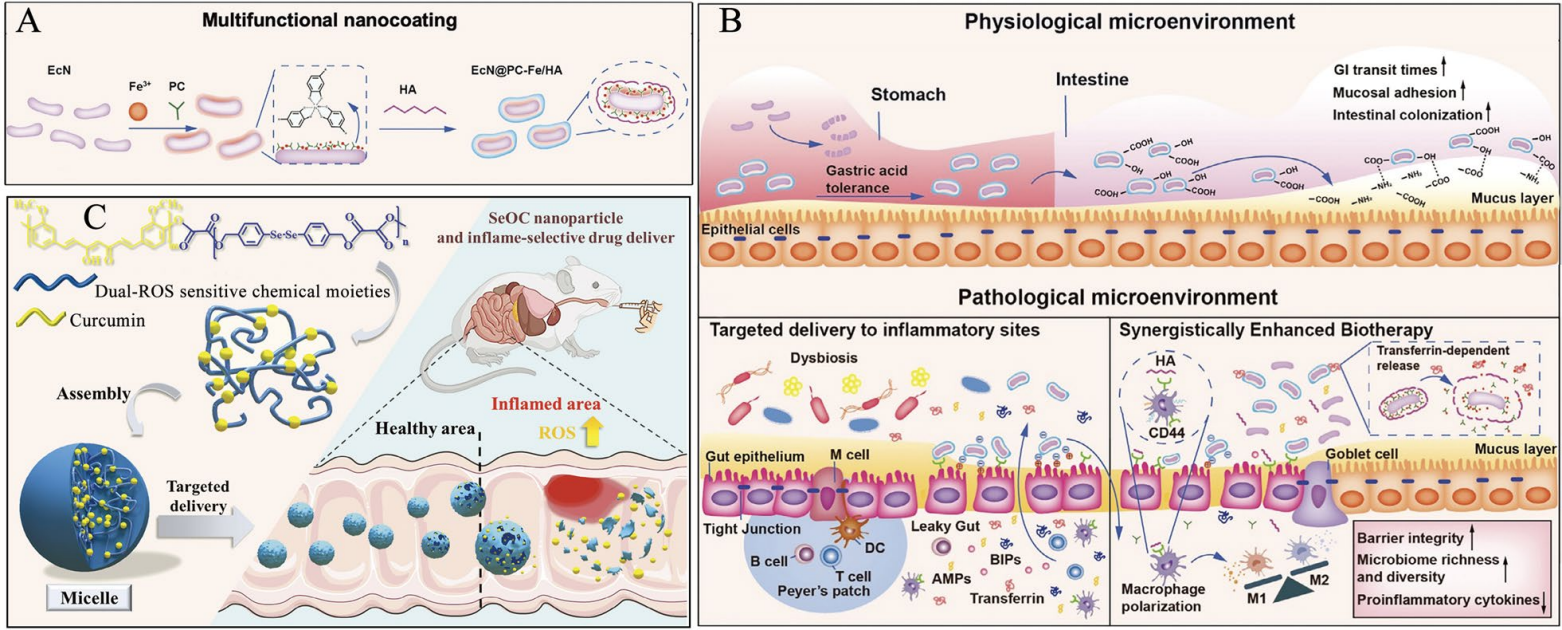
**A** Schematic diagram illustrating the synthetic procedure for EcN@PC-Fe/HA, incorporating PC, Fe^III^, and HA via a layer-by-layer self-assembly strategy [152]; Adapted with permission [152]. Copyright 2024, John Wiley and Sons. **B** The HA-shell improves probiotic survival and intestinal colonization after oral administration of EcN@PC-Fe/HA. Effective inflammatory targeting is achieved through dual actions: HA-CD44 binding and EI. EcN@PC-Fe/HA alleviates intestinal bacterial dysbiosis while inhibiting intestinal barrier damage and acute inflammation, demonstrating synergistic enhancement in treating dextran sulfate sodium-induced colitis [152]; Adapted with permission [152]. Copyright 2024, John Wiley and Sons. **C** Poly(diselenide-oxalate-curcumin) nanoparticles, which incorporate double-ROS-sensitive chemical components (peroxalate ester and diselenide bonds), are prepared using a one-step synthesis strategy. This approach simplifies the fabrication of ROS-responsive nanoparticles, enhancing the oxidation stability of drugs [157]; Adapted with permission [157]. Copyright 2024, John Wiley and Sons. EcN: Escherichia coli Nissle 1917, PC: procyanidine, HA: hyaluronic acid, ROS: reactive oxygen species

The bacterial population in the colon (10^11^–10^13^ CFU/g) is significantly higher than in the stomach (10^3^ CFU/g) and small intestine (10^4^–10^8^ CFU/g) [142]. These colonic microbiota produce enzymes such as nitroreductase, azoreductase, *β*-glucosidase, and cellulase, which can degrade polymers like hyaluronic acid, chitosan, pectin, guar gum, glucose, and sodium alginate [149]—materials that are resistant to breakdown in the stomach and small intestine. By harnessing these microbial activities, several colon-targeted ODDSs have been developed. Hyaluronic acid is particularly valuable for IBD therapy due to its interaction with CD44 on immune cells, which regulates macrophage activation and exerts anti-inflammatory effects [150, 151]. For instance, a hyaluronic acid-functionalized metal-phenolic network (MPN) was used to coat probiotics (Fig. 11A) [152]. The hyaluronic acid layer protected the probiotics from the harsh gastrointestinal environment, including proteases, stomach acids, and bile acids, while improving intestinal barrier homeostasis, reducing pathological inflammation, and restoring intestinal microbiota balance, thus enhancing IBD treatment outcomes (Fig. 11B) [152]. Chitosan, a naturally occurring cationic polysaccharide, exhibits significant properties such as permeability, adhesion, and antibacterial activity [150, 151]. The colon bacterial enzyme N-acetyl-β-D-glucosaminidase specifically hydrolyzes chitosan at the β-(1–4) linkage [149]. Pectin, a complex anionic polysaccharide with a molecular weight of 50–150 kDa found in plant cell walls, resists protease and amylase breakdown but can be degraded by colon bacteria [153]. Pectin with low methylation or long, unbranched oligo-galacturonic acid chains shows strong mucosal adhesion [154]. Guar gum, a natural galactomannan polysaccharide extracted from *Cyamopsis tetragonoloba* [155], has gelling and swelling properties, making it stable across a broad pH range (1.0–10.5) due to its non-ionic and and non-charged nature. It can be decomposed by colon microorganisms such as *Clostridium butyricum* [155] and has bioadhesive and biodegradable properties that protect mucous membranes and alleviate conditions like colorectal cancer [156]. However, due to its high hydration rate, swelling behavior, and susceptibility to microbial contamination, guar gum is often modified before use. For instance, methylation enhances mechanical strength, graft copolymerization reduces biodegradation, and etherification prevents bacterial contamination [149].

### ROS-responsive ODDSs for IBD therapy

**Fig. 12:**
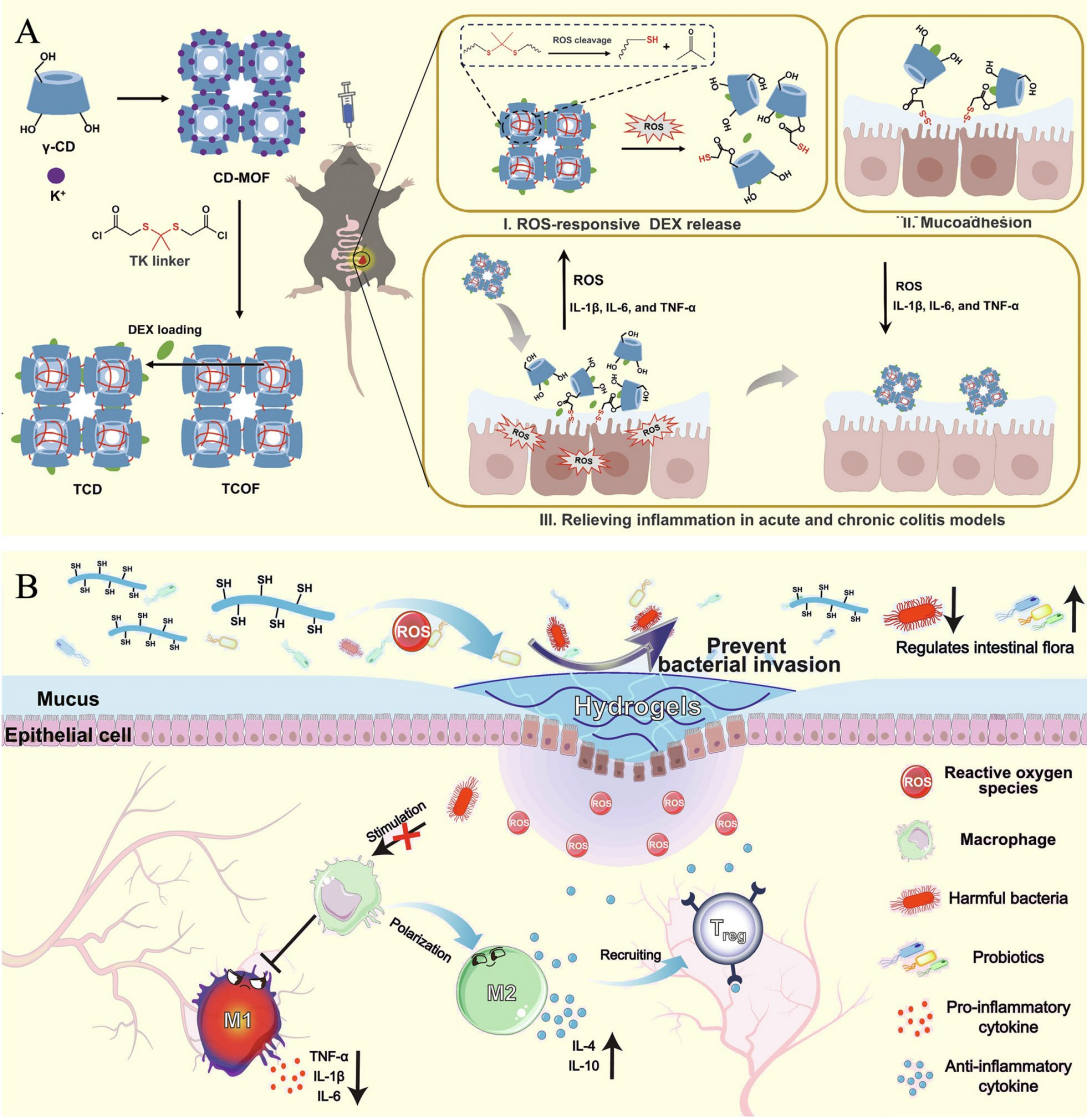
**A** ROS-responsive thioketal (TK)-containing covalently crosslinked frameworks (TCOF) are synthesized using a cubic γ-cyclodextrin-based metal–organic framework (CD-MOF) as the template and thioketal as the crosslinker. Following ROS-induced collapse of TCOF, dexamethasone is selectively released. Dexamethasone-loaded TCOF (TCD) effectively reduces inflammation levels in vitro and protects cells from oxidative stress damage [162]; Adapted with permission [162]. Copyright 2025, John Wiley and Sons. **B** The oral hydrogel precursor solution is primarily composed of sulfhydryl-modified hyaluronic acid. Due to the ROS-responsive property, hydrogel precursor solutions form gels in inflamed areas of the gut, preventing microbial invasion and reducing abnormal immune responses [164]; Adapted with permission [164]. Copyright 2024, The American Association for the Advancement of Science

ROS are one-electron reduction products generated during cellular oxidase metabolism, including superoxide radicals (O_2_^−^), hydroxyl radicals (·OH), singlet oxygen (^1^O_2_), and hydrogen peroxide (H_2_O_2_). ROS serve as key signaling molecules involved in cell signaling and homeostasis regulation in vivo [158, 159]. Under normal conditions, ROS levels remain dynamically balanced. However, during the progression of IBD, abnormally activated immune cells in the affected regions of the colon produce significantly higher concentrations of ROS [160]. This imbalance leads to oxidative stress in the intestinal epithelium, exacerbating the inflammatory response [161]. Clinical studies have shown that neutrophils and macrophages in the intestinal mucosa of patients with IBD generate substantially higher levels of ROS—up to 10 to 100 times more than in healthy individuals [161]. ROS-responsive drug delivery systems offer several advantages: First, they possess inherent ROS-scavenging properties, enabling them to reduce excessive ROS levels. Second, they prevent or minimize the premature release or degradation of the drug before reaching the targeted diseased area. Third, they facilitate the release of the drug in response to elevated ROS levels, delivering high concentrations of the drug specifically to the inflamed regions for optimal therapeutic effects [162]. For example, Liang et al. employed a one-step synthesis strategy to develop poly(diselenide-oxalate-curcumin) nanoparticles incorporating dual-ROS-sensitive components (peroxalate ester bonds and diselenide bonds) (Fig. 11C) [157]. In a colitis model, the dual-ROS-sensitive chemical moieties enabled targeted delivery of curcumin to the colon. In an oxidative microenvironment, the degradation rate of curcumin in the delivery system was approximately four times lower than that of free curcumin, significantly alleviating IBD symptoms and maintaining intestinal microbiota homeostasis [157]. Additionally, Xiong et al. synthesized ROS-responsive thioketal (TK)-containing covalently crosslinked frameworks (TCOF) using a cubic γ-cyclodextrin-based metal–organic framework (CD-MOF) as the template and thioketal as the crosslinker (Fig. 12A) [162]. Dexamethasone, a well-known glucocorticoid with anti-inflammatory properties, was encapsulated within a ROS-responsive thioketal (TK)-containing covalently crosslinked framework (TCOF). Upon ROS-induced degradation of the delivery system, dexamethasone was selectively released. This oral delivery system effectively protected cells from oxidative stress and reduced inflammation, offering therapeutic benefits in chronic colitis [162]. Beyond simply degrading the delivery system, ROS-responsive ODDSs can also induce cross-linking and solidification. For example, ROS can promote the oxidation of mercaptans, leading to the formation of disulfide bonds [163]. A polymer substituted with mercaptan groups can specifically form hydrogels in the presence of ROS at the site of inflammation [163]. Hyaluronic acid, modified with mercaptan groups, was designed to form a ROS-responsive pre-gel system (Fig. 12B) [164]. Due to the ROS-responsive nature of this system, hydrogel precursor solutions form gels specifically in inflamed regions of the gut, providing a barrier against microbial invasion and reducing abnormal immune activation. Furthermore, the hydrogel can regulate the gut microbiota, increasing the abundance of short-chain fatty acid-producing bacteria and promoting greater microbial diversity [164].

### Charge-responsive ODDSs for colonic targeting and therapy

**Fig. 13:**
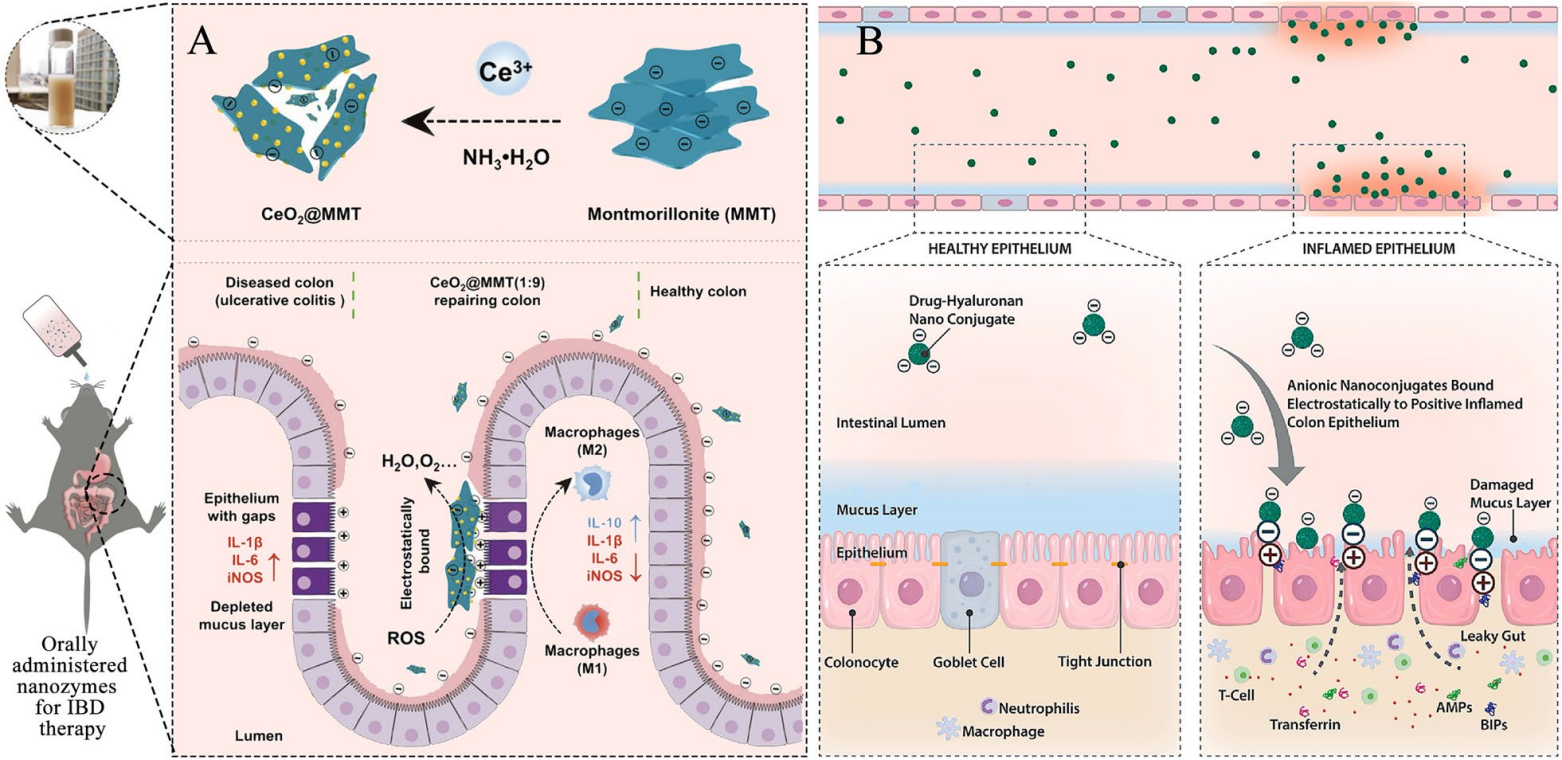
**A** Schematic diagram illustrating the design and synthesis of CeO_2_@Montmorillonite (MMT) for orally administered targeting of inflamed intestines in the treatment of inflammatory bowel disease (IBD). CeO_2_@MMT is synthesized by in situ growth of ceria nanoparticles (CeO_2_ NPs) onto MMT sheets. UC abnormalities include mucus depletion, positively charged protein accumulation, increased intestinal permeability, and elevated levels of pro-inflammatory macrophages and reactive oxygen species. The negatively charged CeO_2_@MMT specifically targets the positively charged inflammatory colon [168]; Adapted with permission [168]. Copyright 2020, John Wiley and Sons. **B** The delivery system, with its anionic surface charge and small size, effectively adheres to and penetrates inflamed mucosa. It shows promising therapeutic effects in dextran sodium sulfate (DSS)- and 2,4, 6-trinitrobenzene sulfonic acid (TNBS)-induced models [169]

The mucus layer of the colon is typically negatively charged. However, at inflamed sites in the colon mucosa, the mucus layer is often depleted, and the inflamed tissue produces large amounts of transferrin and eosinophilic cationic proteins [165]. Other cationic proteins in the inflamed region include antimicrobial anti-proteases, bactericidal β-defensins, and permeability-increasing proteins [166]. Consequently, negatively charged ODDSs or drugs can interact with the positively charged inflamed mucosal areas through EIs, allowing for targeted drug delivery to the colon, enhancing drug retention time, and improving therapeutic efficacy [165]. Research has shown that negatively charged liposomes exhibit twice the adhesion to inflammatory colon mucosa compared to neutral or positively charged liposomes, while on healthy mucosal membranes, positively charged liposomes adhere three times more effectively than neutral or negatively charged liposomes [167]. Zhao et al. utilized negatively charged montmorillonite, coupled with multi-enzyme mimicking CeO_2_ nanoparticles, to create CeO_2_@Montmorillonite for IBD treatment (Fig. 13A) [168]. CeO_2_ displayed CAT-like and superoxide dismutase activity, as well as hydroxyl radical scavenging properties, making it more effective in ROS clearance than non-catalytic antioxidants, while being more stable than free enzymes [168]. When taken orally, negatively charged montmorillonite (with a ζ-potential of about − 30 mV) was specifically adsorbed to the positively charged inflamed tissues via EIs, thus achieving targeted drug delivery [168]. Furthermore, Kotla et al. encapsulated the model drug cyclosporine A within poly(lactic-co-glycolic acid) (PLGA) using layer-by-layer bio-coatings and polymeric nano-drug core emulsification-solvent evaporation methods [169]. The resulting inflammation-specific targeted carriers, with an anionic surface charge (ζ-potential ranging from -30 mV to -45 mV), small size, and mucosal adhesion characteristics, enhanced adhesion and penetration into inflamed mucosa. This system demonstrated strong therapeutic effects in dextran sodium sulfate (DSS) and 2,4,6-trinitrobenzene sulfonic acid (TNBS)-induced models (Fig. 13B) [169]. Additional representative experiments for colon targeting are summarized in Table 1.

### Time-dependent response mode

Time-dependent systems, also referred to as sigmoidal release systems or pulsatile delayed systems, are designed to release drugs after a specific delay [170]. These systems are influenced by the time it takes for the drug to pass through the stomach, which varies significantly between individuals and can also be affected by the patient’s disease condition. This variability makes it difficult to predict the precise timing of drug arrival in the colon. Time-dependent ODDSs must remain intact in the gastric environment (characterized by low pH) and only release the drug once they reach the duodenum, where the pH increases. The release is triggered after a lag phase. The retention time of time-dependent ODDSs in the small intestine is generally constant or experiences minimal variation [171]. Enteric coatings are used to initiate drug release, but the dissolution of these coatings must occur slowly enough to allow the system to pass through the small intestine before targeting the colon. The delayed release typically requires a constant lag phase and reproducible results. Most time-dependent ODDSs are designed for pulsed drug delivery [171]. In the study by Foppoli et al., a time-dependent strategy was employed to prepare a system for colonic delivery of 5-aminosalicylic acid [172]. This system was created by spray-coating the immediate-release ODDSs core with low-viscosity HPMC and Eudragit^®^ L. The enteric coating effectively prevented the release of 5-aminosalicylic acid in the acidic environment, while the HPMC coating introduced a repeatable lag phase before drug release in a phosphate buffer medium. Increasing the thickness of the HPMC coating extended the lag time. This experiment also demonstrated that the transport time in the small intestine was relatively constant and repeatable, aligning with the design principle of time-dependent ODDSs [172]. In a more recent study, Vemula et al. developed colon-specific tablets for flurbiprofen using crude banana peel powder as a novel building block for time-dependent carriers [173]. The structure of the system consisted of crude banana peel as the core, with Eudragit^®^ S 100 coating the surface. Dissolution results indicated that drug release in the upper digestive tract was negligible, with approximately 99% of the drug being gradually released in the colon over 24 h [173]. While time-dependent delivery systems show excellent performance in vitro, there is still limited research on their assessment in the human body [174]. Variations in physiological factors (such as pH, bacterial populations, and transport times), poor reproducibility, and differing pathological conditions in patients may all hinder the widespread application of time-dependent delivery systems in practical clinical settings.

### Pressure-dependent response mode

Peristalsis in the GIT generates pressure within the lumen, which can impact the drug delivery of ODDSs. Intestinal fluid helps reduce the pressure in the small intestine, but in the colon, the reabsorption of water increases the viscosity of the contents. This leads to greater pressure from the peristalsis of the intestinal wall muscles, which grind and push the intestinal contents, resulting in higher pressure in the colon. The formation of feces is evidence of this elevated colonic pressure [175]. Some capsules, tablets, or coated granules may not withstand the pressure generated by colonic peristalsis, causing them to disintegrate and prematurely release the drug. This mechanism forms the basis for the design of pressure-dependent ODDSs. The pressure threshold of these systems can be controlled by adjusting the system's size or the thickness of the shell. Currently, pressure-dependent response systems primarily use capsule and coating technologies to control drug release. Some pressure-dependent ODDSs feature insoluble surface coatings, such as polyethylene glycol and stearic acid [122, 176]. Additionally, ethyl cellulose, a common film-coating material and tablet adhesive, is widely used due to its non-toxicity, stability, and insolubility in water [177]. In an experiment by Muraoka et al., pressure-controlled colon delivery capsules were prepared by coating ethyl cellulose onto the inner surface of gelatin capsules [177]. The hardness of the capsules could be controlled by adjusting the thickness of the ethyl cellulose coating. Driven by the physiological pressure within the lumen, the capsule disintegrates in the intestinal tract. Capsules with an average coating thickness of 50 μm and hardness of 2.08 Newtons were shown to effectively transport caffeine to the colon for controlled release [177]. Similarly, Jeong et al. developed pressure-dependent colonic delivery capsules using the impregnation method [178]. The capsule consisted of two polymer membranes, with the inner layer being water-insoluble and the outer layer being enteric-coated. Fluorescein was used as a model drug. Compared to the colon arrival times of 3.5 h, estimated by a sulfasalazine test, pressure-dependent ODDSs delivered fluorescein to the colon, with the drug reaching the systemic circulation between 3.5 and 3.8 h. This preparation method is particularly suitable for mass production in capsule manufacturing, facilitating the rapid development of colonic drug delivery systems [178]. Furthermore, pressure-dependent colonic delivery capsules can also be prepared by coating sealed-hard gelatin capsules with Eudragit S100-based materials, as demonstrated in the treatment of nocturnal asthma with theophylline. This system exhibited reduced pharmacokinetic variability in vivo compared to commercially available formulations [179]. While pressure-dependent ODDSs are safe and effective, their delivery performance may vary among individuals due to the influence of disease states, food intake, and excretion, all of which affect colonic lumen pressure.

### Combination systems for colonic targeting and therapy

Considering the variability and individual differences in the physiological environment of the colon, relying on a single response mode may not achieve the desired drug delivery efficiency. For instance, pH-dependent ODDSs for colon-targeted delivery may release the drug prematurely in the small intestine or fail to release it in the colon due to the lack of specific targeting site characteristics [180]. Similarly, in time-dependent systems, the timing of drug release largely depends on the duration it takes for the ODDSs to pass through the stomach. Variations in food content and individual differences result in inconsistencies in gastric transit times, which can cause premature drug release in the small intestine or delay drug release until the distal colon [180]. Thus, there has been growing interest in combining different response mechanisms to develop more effective delivery systems [146]. These systems are categorized into parallel-triggered and sequentially triggered systems based on their triggering mechanisms [146]. In parallel-triggered systems, multiple mechanisms exist independently and are activated once their respective thresholds are reached, ensuring that if one mechanism fails to trigger, others can compensate to ensure targeted release. In sequentially triggered systems, the drug release occurs in stages, with the outermost layer triggering first and the subsequent internal layers being activated in sequence to release the drug [122]. In the context of multi-response systems, Sardou et al. explored this concept extensively [181–183]. They developed 5-aminosalicylic acid matrix pellets using the extrusion-spheronization method, achieving a drug loading rate of 70%. By optimizing the coating formulations of Eudragit S, Eudragit L, and Ethylcellulose, they created a system that delivered the drug to the colon via both pH- and time-dependent mechanisms. This delivery system demonstrated more precise drug targeting to the colon compared to commercial products like Asacol^®^ and Pentasa^®^ [181]. Moreover, given the vast number of intestinal microbiota in the colon, incorporating a microbial response mechanism into the delivery system has shown promising results. Shafaei et al. combined microbial response-dependent systems with pH-dependent systems to develop an innovative colonic drug delivery system [182]. This system was prepared using various polysaccharides (xanthan, inulin, carrageenan, and alginate) and Eudragit FS 30 D as raw materials via the film formation method. Their findings revealed that inulin film was an ideal substrate for colonic drug delivery, owing to its suitable mechanical properties, low expansion rate, and sensitivity to colon-specific enzymes [182]. Additionally, enteric- and time-dependent systems have also demonstrated effective performance in colon-targeted delivery [183].

## Clinical trials

Currently, ODDSs face significant challenges in transitioning from laboratory settings to clinical applications, with only a small number of products advancing to the clinical trial stage [175]. Ongoing or completed clinical trials are summarized in Table 2 [175]. Much of the existing knowledge about ODDSs has been derived from animal models. However, the human body is a far more complex system, and species differences often complicate the accuracy of predicting therapeutic outcomes in clinical trials [184]. Additionally, many nanoscale carriers have not been comprehensively assessed for systemic and local toxicity in normal tissues. For example, some carriers can interact with the immune system, potentially triggering allergic or hypersensitive reactions [185]. Others may induce changes in cell morphology, disrupt intracellular signaling pathways, and lead to adverse biological interactions [184]. These issues are often discovered during clinical trials, which explains why many carriers fail to progress in clinical research. Moreover, more complex carriers typically require multi-step synthesis processes, complicating large-scale production. In contrast, simpler and more accessible systems tend to have a higher likelihood of reaching clinical use. Researchers are currently conducting numerous clinical trials in hopes of bringing these delivery systems to the clinic, thereby benefiting patients [186]. In one clinical trial (NCT02291445), a novel peppermint oil formula was studied for its ability to gradually release peppermint oil in the colon [187]. The aim was to enhance therapeutic efficacy while minimizing side effects. The trial also assessed pharmacokinetic characteristics, safety, and tolerability, comparing the new formulation with commercially available peppermint oil products. Another Phase I clinical trial (NCT03774680) is evaluating a new agent for colorectal cancer treatment [188]. This formulation, which utilizes ethyl cellulose polymer, is designed to load the anti-cancer drug cetuximab and release it when the pH exceeds 6.8. The formulation has demonstrated the ability to be safely administered in large doses, targeting tumor cells with minimal side effects on healthy tissues.

**Table 2:** Representative clinical trials of oral gastrointestinal targeted delivery systems

Diseases	Characteristics	Clinical status	National clinical trial number
Ulcerative colitis	Colon microbiota activated COLAL-PRED^®^ system	Completed	NCT00299013
Colon cancer	pH-responsive and cell-targeted release Cetuximab	Phase I	NCT03774680
Type 1 diabetes	Silica nanoparticles delivering insulin orally	Phase II	NCT01973920
Type 2 diabetes	Mucus penetrated tablet, a co-formulation of GLP-1 RA with SNAC	Completed	No available
Irritable bowel syndrome	Colon-targeted-delivery Peppermint Oil Capsule	Completed	NCT02291445
Antibacterial activity	Colon-targeted delivery of Brilacidin	NO available	NCT04240223
Insomnia	Gastric expandable systems to release Zaleplon	Phase II	NCT01277107

## Challenges and future perspectives

The field of ODDSs is poised for rapid growth, particularly with patient-centered designs that enable more targeted administration and enhance cost-effectiveness. However, several challenges remain, which also represent key areas for future development to facilitate the broader application of ODDSs.

1. The stability of ODDSs requires precise control. Current systems typically consist of various building blocks assembled through non-covalent or covalent interactions. If these interactions result in excessive stability, drug release at the target site may be hindered. On the other hand, insufficient stability may lead to premature drug leakage before reaching the intended destination.
2. Few ODDSs are capable of overcoming the biopotency barriers of the GIT. These barriers are complex and include factors such as acidic and enzymatic degradation, poor stability, first-pass metabolism, and premature systemic absorption from the stomach or small intestine [189]. Each additional barrier crossed significantly reduces the likelihood of successful drug delivery. To address this, cascaded targeting and multi-stimulus response delivery platforms have been developed to improve localization after absorption into the blood or lymphatic system [190, 191].
3. A deeper understanding of the physiological properties of the gastrointestinal biological barrier is essential. Currently, most ODDS design methods aimed at overcoming the GIT barrier are similar, and new mechanisms need to be explored. Li et al. discovered that CAT expression was higher in the small intestine than in other parts of the GIT, such as the stomach and colon. This finding led to the development of the GSEL method to specifically target the small intestine [57].
4. The targeting ability of ODDSs remains inferior compared to injectable delivery systems. Targeting can be categorized into three levels [192]. The first level involves targeting specific tissues or organs, while the second level targets specific cells, such as the parenchymal cells in liver tissue affected by liver cancer, rather than non-parenchymal cells (e.g., Kupffer cells, endothelial cells) [193]. The third level involves intracellular targeting, where the goal is to reach specific organelles within a cell [192]. Injectable drug delivery systems, which bypass the gastrointestinal tract, can reach the second or even third level of targeting after injection. In contrast, ODDSs face the significant challenge of crossing the GIT barrier and, once in the bloodstream or lymphatic system, targeting additional sites. Consequently, most current ODDSs are confined to achieving only the first level of targeting.
5. A standardized protocol for evaluating the performance of ODDSs needs to be established. The fate of ODDSs in the body is highly complex, making their evaluation challenging. Over the years, various methods have been developed to assess the performance of drug delivery systems, including evaluations of interactions, stability, retention/release mechanisms, absorption pathways, bioavailability, and transport behavior [194, 195]. However, to accurately assess the efficacy of these systems in specific applications, it is essential to create standardized evaluation methods. This will facilitate the identification of the optimal protocols for delivering drugs with varying properties.
6. The safety of ODDSs must undergo comprehensive evaluation. Due to their complexity compared to injectable delivery systems, ODDSs involve a broader range of building blocks, some of which or their combinations may pose toxicity risks. For instance, Hou et al. prepared nanoparticles using polyethyleneimine (PEI) and PLA to encapsulate the hydrophobic drug paclitaxel [196]. PLA was found to be non-toxic, while PEI exhibited some degree of toxicity.
7. The development of oral multi-drug co-delivery systems is needed. Most ODDSs are currently designed to deliver a single drug. However, the co-delivery of two or more drugs with distinct physiological mechanisms could offer the potential for synergistic disease treatment.
8. Individualized treatment and customized ODDSs are essential. Gastrointestinal conditions vary significantly between patients, with differences in disease progression that may require ODDSs with tailored release rates and drug dosages.
9. The integration of diagnosis and treatment represents a long-term goal for ODDSs. The combination of drug delivery, imaging, and real-time monitoring of disease progression and treatment effectiveness could pave the way for more precise and personalized therapeutic strategies.
10. The use of artificial intelligence (AI) can significantly accelerate the development of ODDSs. With the rapid advancements in AI over recent years, large models such as Generative Pre-trained Transformer (GPT) and DeepSeek have reached unprecedented levels of capability. AI-based models can simulate the distribution, metabolism, and excretion of ODDSs within the body. These models can predict the physicochemical stability of ODDSs in the gastrointestinal barrier, estimate drug loading efficiency, and simulate release rates, among other factors [197, 198]. Additionally, by leveraging AI’s strengths in data analysis, pattern recognition, and optimization, ODDS developers can improve patient outcomes, reduce side effects, and enhance treatment efficacy. AI is revolutionizing ODDSs by enabling more personalized, customized, and streamlined approaches [199, 200].
11. The transition of ODDSs from laboratory research to clinical application is critical. Several factors must be addressed for the commercialization of ODDSs, including consumer acceptance, robustness, labeling, sustainability, affordability, environmental friendliness, regulation, and safety [201]. To date, most ODDSs developed in laboratory settings have proven unsuitable for large-scale industrial production. For instance, Luan et al. developed “wooden rolls” designed to deliver bioactive substances and probiotics orally [202]. However, their relatively large size has posed challenges, including difficulties with swallowing, unpleasant taste, and psychological resistance from consumers.

## Conclusions

ODDSs are increasingly favored by patients due to their non-invasive, needle-free nature, offering a more cost-effective and time-efficient alternative to invasive treatments. This review initially explored key gastric targeting strategies aimed at increasing retention time in the stomach. The primary principle involves manipulating the density and geometry of ODDSs to prevent premature gastric emptying. For small intestine targeting, pH-dependent methods are commonly employed, with the GSEL method being a recent development. To enhance interaction with and penetration through the mucus layer, EIs are often used to target the intestinal mucus. Transepithelial transport occurs via three main pathways: paracellular transport, transcytosis, and the M cell-associated pathway. Methods to improve epithelial cell targeting and enhance absorption include opening TJs and modifying surface ligands on ODDSs. In terms of colon targeting and IBD treatment, ODDSs leverage mechanisms such as more alkaline pH, diverse intestinal flora, ROS-responsiveness, and charge-responsiveness. The significant advancements in life sciences over the past decade have made the widespread adoption of ODDSs more feasible than ever. Thus, the future of oral administration is poised for rapid evolution, offering new treatment possibilities for both patients and clinicians. 

## Data Availability

No datasets were generated or analysed during the current study.
